# Zika virus tropism during early infection of the testicular interstitium and its role in viral pathogenesis in the testes

**DOI:** 10.1371/journal.ppat.1008601

**Published:** 2020-07-02

**Authors:** Konstantin A. Tsetsarkin, Joshua A. Acklin, Guangping Liu, Heather Kenney, Natalia L. Teterina, Alexander G. Pletnev, Jean K. Lim

**Affiliations:** 1 Laboratory of Infectious Diseases, National Institute of Allergy and Infectious Diseases (NIAID), National Institutes of Health (NIH), Bethesda, Maryland, United States of America; 2 Department of Microbiology, Icahn School of Medicine at Mount Sinai, New York, New York, United States of America; 3 Graduate School of Biomedical Sciences, Icahn School of Medicine at Mount Sinai, New York, New York, United States of America; Florida State University, UNITED STATES

## Abstract

Sexual transmission and persistence of Zika virus (ZIKV) in the testes pose new challenges for controlling virus outbreaks and developing live-attenuated vaccines. It has been shown that testicular infection of ZIKV is initiated in the testicular interstitium, followed by spread of the virus in the seminiferous tubules. This leads to testicular damage and/or viral dissemination into the epididymis and eventually into semen. However, it remains unknown which cell types are targeted by ZIKV in the testicular interstitium, and what is the specific order of infectious events leading to ZIKV invasion of the seminiferous tubules. Here, we demonstrate that interstitial leukocytes expressing mir-511-3p microRNA are the initial targets of ZIKV in the testes, and infection of mir-511-3p-expressing cells in the testicular interstitium is necessary for downstream infection of the seminiferous tubules. Mir-511-3p is expressed concurrently with CD206, a marker of lineage 2 (M2) macrophages and monocyte derived dendritic cells (moDCs). Selective restriction of ZIKV infection of CD206-expressing M2 macrophages/moDCs results in the attenuation of macrophage–associated inflammatory responses *in vivo* and prevents the disruption of the Sertoli cell barrier *in vitro*. Finally, we show that targeting of viral genome for mir-511-3p significantly attenuates early ZIKV replication not only in the testes, but also in many peripheral organs, including spleen, epididymis, and pancreas. This incriminates M2 macrophages/moDCs as important targets for visceral ZIKV replication following hematogenous dissemination of the virus from the site of infection.

## Author summary

Zika virus (ZIKV) has recently gained global media attention as a major emerging pathogen. Unique among the flaviviruses, ZIKV can be transmitted sexually as well as through the canonical arthropod route. Transmission through sexual contact raises new challenges for controlling ZIKV outbreaks, and infection of the testes poses a unique challenge for the development of live attenuated vaccines against ZIKV. In this manuscript, we utilized the microRNA targeting technique to demonstrate that testicular macrophages/DCs are a critical first target for ZIKV, and infection of these cells is required for passage of the virus through the Sertoli cell barrier and subsequent infection of the seminiferous tubules. Our investigations are the first to clearly show the sequence of infection events required for testicular infection that would subsequently lead to sexual transmission of ZIKV.

## Introduction

Zika virus (ZIKV) is a positive sense RNA flavivirus that was originally isolated from a rhesus macaque in 1947 in Uganda. Following its discovery, ZIKV remained a rather obscure pathogen, with infections primarily resulting in a mild self-limiting febrile illness. However, during these decades, the virus spread from Africa to South Asia, then across the Pacific to the Yap Islands (reviewed in [1]). It wasn’t until its introduction into the New World that ZIKV was associated with severe symptomatology in pregnant women, with microcephaly among the most devastating outcomes [2]. Indeed, according to the CDC, 5–10% of infections that occur during pregnancy are associated with congenital Zika syndrome, and this parameter can reach as high as 15% among first trimester infections [3]. Given the explosive nature of the recent outbreak, there has been a major push for the development of safe, efficacious vaccines to prevent future ZIKV outbreaks, particularly to protect pregnant women. One major hurdle for the development of any ZIKV vaccine strategy is to consider the testicular tropism of the virus and its implication for sexual transmissibility (reviewed in [4, 5]). In mice, ZIKV infection of the testes has been associated with testicular swelling and male infertility [6–8]. In humans, the impact of testicular ZIKV infection is unclear; however, viral RNA can be found in semen for up to several months after initial infection, and infectious particles have been found for up to 69 days post infection ([9–15]; reviewed in [5, 16]). In this regard, a safe attenuated vaccine strategy should avoid testicular replication in humans without compromising immunogenicity.

One strategy to achieve selective restriction of RNA virus replication in specific cells or tissues is through microRNA (miRNA) targeting, which is a convenient tool for modulating viral tropism. We recently characterized a ZIKV clone 2×scr [17], which contains two copies of scrambled (scr) sequences (20 nucleotide (nt) each) inserted after nt positions 8 and 14 of the 3’ noncoding region (3’NCR) of the ZIKV-NS3m infectious cDNA clone [Asian lineage, strain Paraiba_01/2015] [18]. These scr sequences can be replaced with functional targets for any miRNA expressed by the host, generating miRNA-targeted ZIKV clones. With careful miRNA target selection, this can be utilized as a powerful tool to modulate ZIKV tropism *in vitro* and *in vivo*.

Following infection of male AG129 mice with ZIKV clone 2×scr, we previously showed that there were two separate peaks of viral infection in the testes, which occurred 3 and 12 days post infection (dpi), respectively. At 3 dpi, the 2×scr virus infected cells localized in the testicular interstitium, while at 12 dpi, virus was primarily found in the cells of the seminiferous tubules, suggesting a temporal regulation of viral tissue replication or tropism [17]. Similar kinetics of viral replication and pathogenesis in the testes was observed for natural isolates of ZIKV following infection in mice that have various deficiencies in innate immune signaling pathways [6–8, 19–21]. We also showed that insertion of target sites for miRNAs that are selectively expressed in the cells located inside the seminiferous tubules of the mouse testes (i.e. mir-202-5p or mir-465a-3p) resulted in the inability of ZIKV to replicate only during the late phase of infection (12 dpi peak) but had no effect on early replication, which peaks at 3 dpi [17]. These observations, together with a previous study regarding the role of testicular inflammation in ZIKV pathogenesis [22], suggest that early replication in the testicular interstitium may be an important step for productive infection of the seminiferous tubules. Uncontrolled ZIKV infection of the seminiferous tubules leads to testicular damage and viral dissemination into the epididymis in mice [17], from where the virus can be secreted into the semen, thus accounting for the sexual transmissibility of ZIKV.

Currently, it remains unknown what cell type is primarily targeted by ZIKV in the testicular interstitium, or what is the temporal sequence of events that precedes ZIKV invasion into seminiferous tubules. The testicular interstitium harbors several types of cells, including testosterone producing Leydig cells, peritubular myoid cells, fibroblasts, endothelial cells, macrophages, dendritic cells (DC), T-lymphocytes and mast cells [23, 24]. It was recently shown that primary human Leydig cells fail to support efficient ZIKV replication in cell culture [25, 26]. In contrast, studies of IFN-receptor deficient mice and human testicular explants demonstrated that ZIKV can infect macrophages, which constitute the most abundant group of testicular leukocytes [23, 27], rather than myoid or Leydig cells [19, 28]. These studies suggest that testicular macrophages may be the primary target for early ZIKV infection in the testicular interstitium. Macrophages are a diverse population of monocyte-derived cells, generally classified into classically (M1) and alternatively (M2) activated/polarized cells. M1 macrophages are induced by pro-inflammatory stimuli such as TNF-α, IFN-γ or lipopolysaccharide, while M2 macrophages are activated in response to anti-inflammatory cytokines such as IL-4, IL-10, IL-13 and TGF-β (reviewed in [29]). It has been shown that the majority of tissue resident macrophages in the testes are M2 macrophages [30, 31]. These M2 macrophages are delineated into interstitial and peritubular. These two types of macrophages play important roles in the normal homeostasis of the testes (reviewed in [32]). We therefore hypothesized that M2 macrophages may be important early targets for ZIKV replication in testes as well as in other peripheral organs.

To our knowledge, there is no single miRNA that is selectively expressed in all populations of macrophages. However, it has been shown that M2-polarization of macrophages is associated with robust activation of CD206 gene (also known as a mannose receptor gene 1 (MRC1)) expression, which is induced by GM-CSF, IL-4, IL-10, IL-13 and TGF-β. Besides M2 macrophages, CD206 is expressed in monocyte-derived dendritic cells (moDCs) and at low levels in M1 macrophages, but not in undifferentiated monocytes [33]. Interestingly, the fifth intron of CD206 gene encodes a pre-miRNA molecule that is processed by the cellular miRNA machinery to produce mature mir-511-3p miRNA [34]. This miRNA is expressed concurrently with CD206 mRNA from the common pre-mRNA [35–37]. Thus, we reasoned that mir-511-3p could be a suitable target for the selective restriction of viral replication in testicular M2 macrophages and moDCs among the cell types present in the testes.

Here, we demonstrate that interstitial macrophages and/or moDCs expressing mir-511-3p are the initial targets of ZIKV in the testes during the early course of infection *in vivo*. Infection of mir-511-3p-expressing cells in the testicular interstitium is a prerequisite of downstream ZIKV infection of the seminiferous tubules. We show that selective restriction of ZIKV infection in CD206-expressing macrophages causes both an attenuation of macrophage-associated inflammatory responses *in vivo* and an inability to disrupt the Sertoli cell barrier (SCB) *in vitro*.

## Results

### Targeting of ZIKV genome for Macrophage/DC-specific miRNA restricts ZIKV invasion of the testes

**Fig 1:**
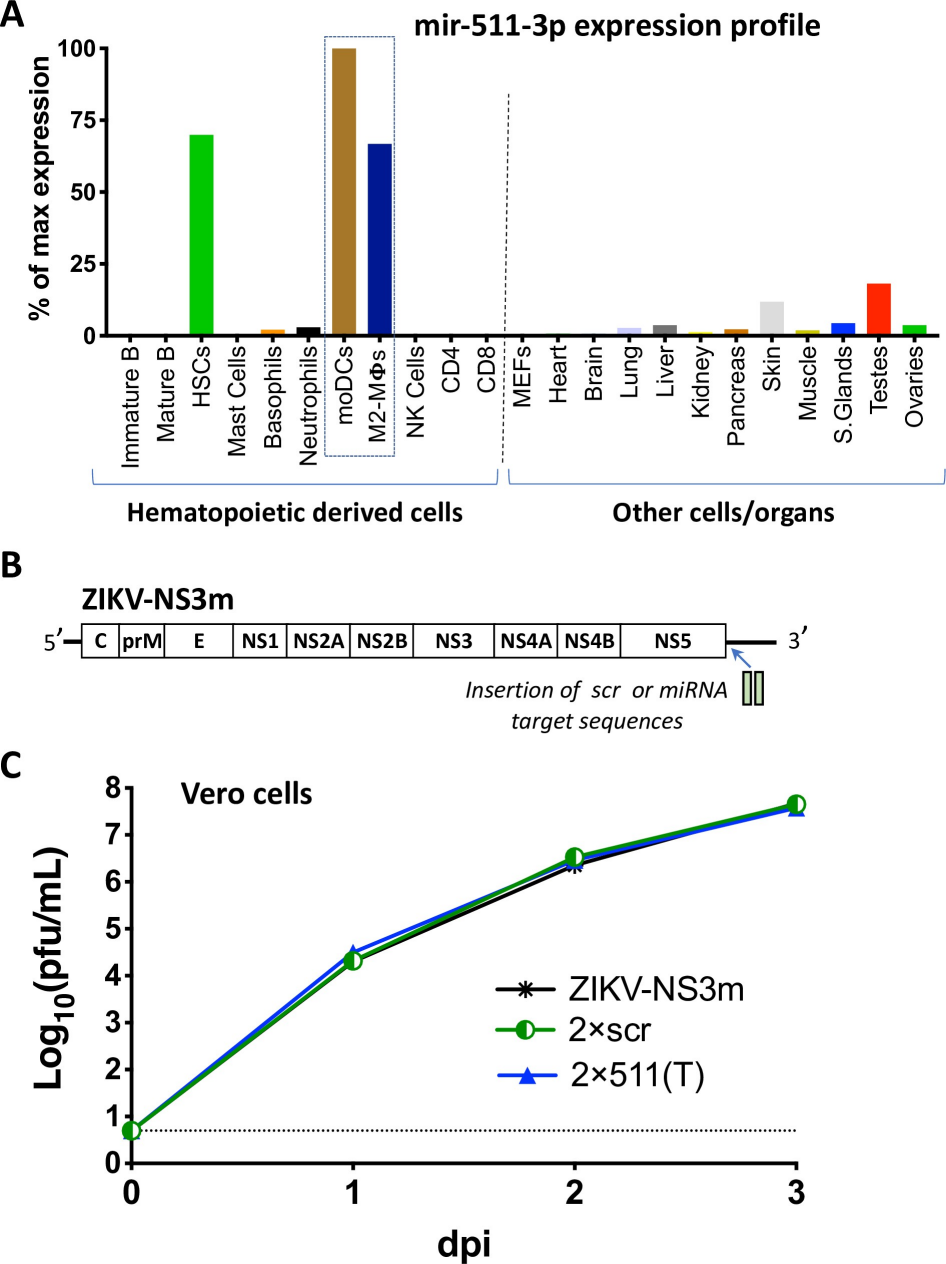
Characterization of a ZIKV clone containing target sites for M2 macrophage/moDC-specific miRNA. **(A)** Expression profile of the mir-511-3p in selected cells and organs of mice. The graph was constructed based on deep sequencing data of the mouse miRNAs previously reported [38]. The expression profile is presented as a proportion of the number of reads for mir-511-3p in the cells/organs to the number of reads for this miRNA in the moDCs, which has the highest expression level among all types of cells or organs. HSCs—hematopoietic stem cells, MΦs-macrophages, NK—natural killer, MEFs–mouse embryonic fibroblasts. (**B**) Schematic representation of the insertion of scr or mir-511-3p target sequences into the genome of ZIKV-NS3m virus [18]. (**C**) Growth of ZIKV-NS3m, 2×scr, and 2×511(T) viruses in Vero cells after plasmid DNA transfection. Data show mean virus titer ± standard deviation (SD) in cell culture supernatants, which were collected daily from duplicate flasks of transfected Vero cells. The dashed line indicates the limit of virus detection [0.7 log_10_(pfu/mL)].

To investigate the role of testicular M2-macrophages in ZIKV infection of the testes, we selected mir-511-3p miRNA for ZIKV genome targeting experiments. This miRNA is predominantly expressed in human and mouse M2-activated macrophages and moDCs (**Fig 1A**). It is also detected in the hematopoietic stem cells, but not in other types of leukocytes [34, 38]. Importantly, mir-511-3p is not expressed in monocytes (see [34] for details) or neutrophils, blood cells previously shown to be peripherally infected by various flaviviruses, including ZIKV [39–41]. This suggests that insertion of targets for mir-511-3p into the ZIKV genome should not interfere with the ability of the virus to spread initially from the site of infection, thus making it an attractive candidate for restriction of testicular M2 macrophages/moDCs infection by ZIKV. Among mouse organs, mir-511-3p was most abundantly expressed in the skin and testes, while it was almost absent in the brain and heart (**Fig 1A**).

To generate a ZIKV clone with restricted capacity to replicate in M2 macrophages/moDCs, we inserted two complementary target (T) sites for mir-511-3p into the 3’NCR of ZIKV-NS3m, which we refer to as 2×511(T). As a control, we used the previously described 2×scr virus, which contained two copies of a scrambled sequence inserted into the 3’NCR (**Fig 1B** and **S1 Fig**). Compared to the parental ZIKV-NS3m strain, both the 2×511(T) and 2×scr viruses replicated with similar growth kinetics in Vero cells (**Fig 1C**), demonstrating that a small sequence insertion into the 3’NCR of ZIKV does not cause non-specific reduction in replicative fitness of the virus in the cells lacking the expression of complementary miRNAs.

**Fig 2:**
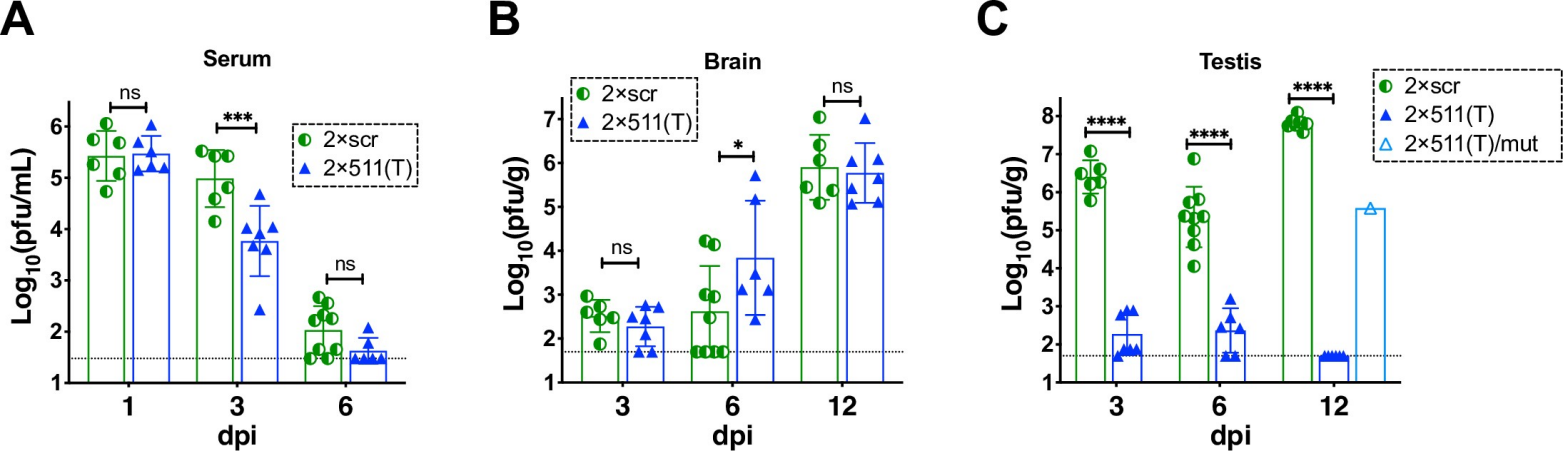
Effect of mir-511-3p target insertions on ZIKV replication in the serum, brain and testes of AG129 mice. Adult AG129 male mice (n = 6–9 per group) were infected IP with 10^6^ pfu of ZIKV clones containing target sites for mir-511-3p [2×511(T)] or scrambled target sites [2×scr]. Mice were bled and/or sacrificed at the indicated days post infection. Mean viral titer ± SD in the serum (**A**), brain (**B**), or testes (**C**) was determined by titration in Vero cells. The dashed lines indicate the limit of virus detection: 1.5 log_10_ pfu/mL of serum (**A**) and 1.7 log_10_ pfu/g of brain or testis (**B-C**). Differences between the virus titers were compared using two-way ANOVA (**** p < 0.0001; *** p< 0.001; * p<0.05; ns—denotes not significant, p> 0.05). Except 6 dpi, all data for the 2×scr virus are retrospective [17] and provided here for comparison. Data for 6 dpi for the 2×scr virus was combined from the data reported earlier (n = 6) [17] and newly generated results (n = 3). Viral RNA was isolated from the serum at 1 dpi, brain at 12 dpi and testes at 12 dpi, and the region containing miRNA targets was sequenced. If mutation/deletion was detected in the miRNA targeted region, viral titer for these samples was reported under the name 2×511(T)/mut.

Natural isolates of ZIKV do not replicate efficiently in mice that have an intact interferon (IFN) signaling pathway (reviewed in [42]). Therefore, all animal studies were conducted using the highly permissive adult (4–6 week old) type I and type II IFN receptor gene knockout AG129 mice [17, 43–45]. Mice infected intraperitoneally (IP) with 10^6^ pfu of 2×scr or 2×511(T) virus developed similar viremia on 1 dpi, although clearance of the 2×511(T) virus from the mouse serum occurred faster compared to that of 2×scr (**Fig 2A**). Sequence analysis showed that all mir-511-3p targets in 2×511(T) and scr sequences in 2×scr isolated from the serum of all mice on 1 dpi remained stable. Next, we evaluated viral titers in the brains of mice infected with 2×scr or 2×511(T) virus at 3, 6, and 12 dpi. As shown in **Fig 2B**, both viruses attained similar titers in the brain at 3 and 12 dpi, and the brain titer of 2×511(T) was slightly higher than that of 2×scr at 6 dpi. Moreover, mir-511-3p targets in the 2×511(T) virus isolated from the brain of 100% mice (n = 7) at 12 dpi remained stable. Together, these data indicate that selective restriction of ZIKV replication in M2 macrophages/moDCs does not impact the capacity of ZIKV to disseminate from the site of inoculation, nor does it limit the capacity of the virus to replicate in the central nervous system (CNS).

In contrast to the effects on ZIKV replication in the brain and serum, restricting ZIKV replication in M2 macrophages/moDCs prevented the accumulation of ZIKV in the testes of AG129 mice. Compared to 2×scr, the mean titer of the 2×511(T) virus was significantly reduced during the early (3 dpi), middle (6 dpi) and late (12 dpi) phase of testicular infection (**Fig 2C**). This suggests that ZIKV infection of M2 macrophages/DCs during the early phase of testicular infection represents a critical step in ZIKV progression to the late phase infection, which is typically associated with robust ZIKV proliferation in the seminiferous tubules (see **S2 Fig** and [17]). Only one out of seven mice infected with 2×511(T) had a moderate virus titer in the testes at 12 dpi. Sequence analysis of the 2×511(T) virus RNA, which was isolated from this mouse at 12 dpi, identified a 40 nt deletion affecting both copies of mir-511-3p target, which was present only in the testis-derived sample, but not in the brain or serum sample (**S3 Fig**). These data further strengthen the role of mir-511-3p-expressing cells as critical targets of ZIKV infection in the testis, as the presence of these 40 nt was selected against in the testes, but not in the CNS.

### Targeting of ZIKV genome for mir-511-3p prevents infection of macrophages/DCs in testicular interstitium

**Fig 3:**
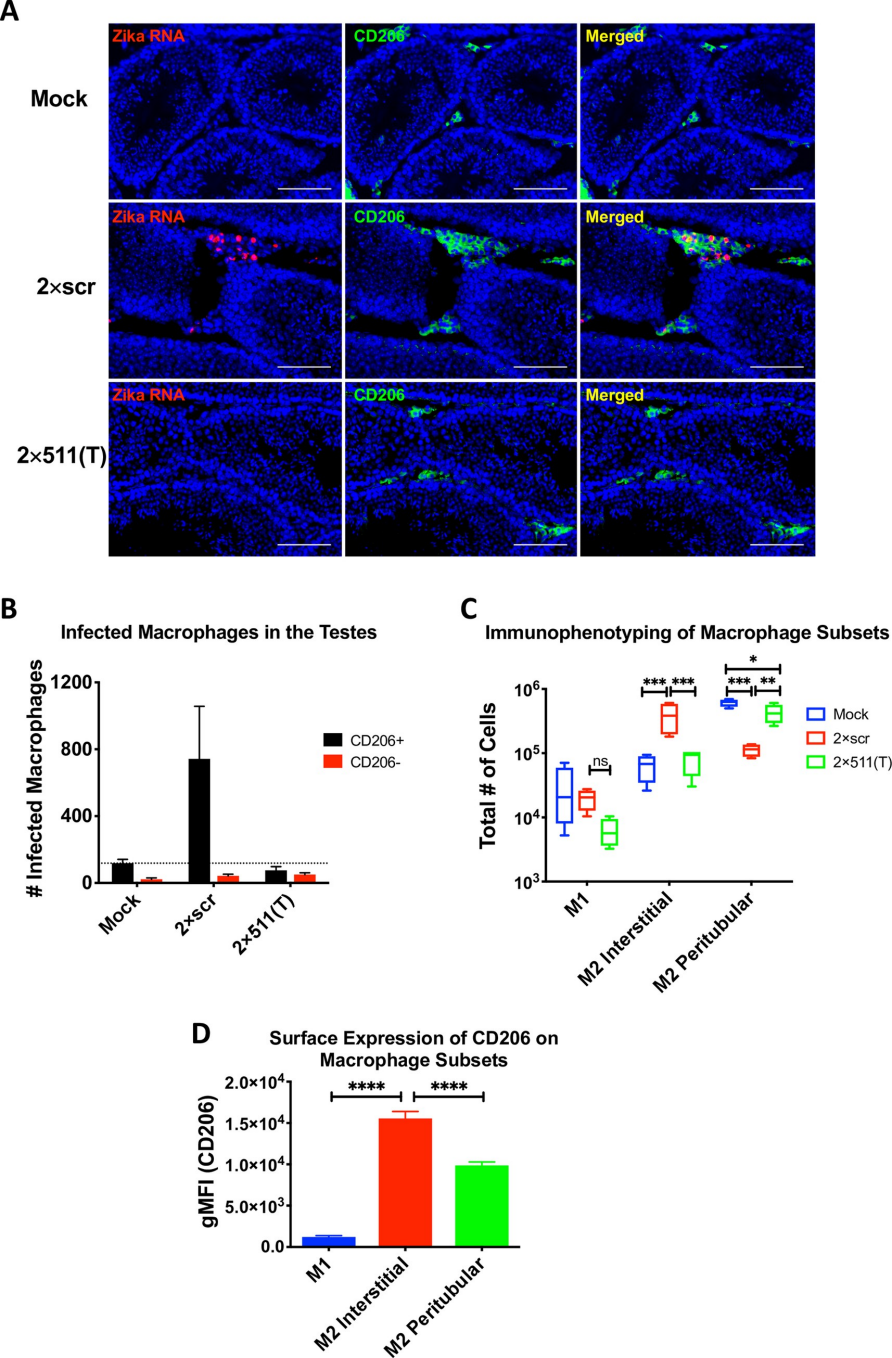
Targeting of ZIKV genome for mir-511-3p prevents infection of CD206 expressing macrophages in the testicular interstitium. Adult AG129 male mice were mock-inoculated or infected IP with 10^6^ pfu of 2×scr or 2×511(T) virus. For panel (**A)**, mock (n = 1), 2×scr (n = 3) and 2×511(T) (n = 3); for panels (**B-D),** mock (n = 3), 2×scr (n = 3) and 2×511(T) (n = 4). Mice were sacrificed at 3 dpi and testes were harvested. **(A)** Testes sections stained for ZIKV RNA (red) by *in situ* hybridization and CD206 (green) by immunofluorescence co-staining. Scale bars represent 100μm. To identify the ZIKV-infected cells within the CD206-positive and CD206-negative populations, cells from testes at 3 dpi were dissociated into a single cell suspension, fixed, and stained for ZIKV using anti-E protein antibody, 4G2, to detect ZIKV and CD206 (**B**). Single cell suspensions from testes were stained with CD45, F4/80, CD11c, CD11b, MHC II and CD206 to differentiate various myeloid cell types (**C**). (**D**) Expression of CD206 was determined in each cell population identified in **C**. Statistical significance was determined for (**C**) and (**D**) by two way ANOVA and one way ANOVA for multiple comparisons. *, **, ***, **** indicate p<0.05, p<0.01, p≤0.001 and p≤0.0001, respectively.

To evaluate the role of testicular macrophages during the early phase of testicular infection, we analyzed the distribution of ZIKV infected cells in the testes of AG129 mice that were infected IP with 10^6^ pfu of 2×scr or 2×511(T) virus. On day 3 post infection, we evaluated testes tissues using RNA *in situ* hybridization to detect ZIKV and antibody co-staining for macrophage markers CD206 and Mac2. Compared to mock-infected mice, we found ZIKV RNA positive cells primarily in the testicular interstitium (**Fig 3A**), but not in the seminiferous tubules of the testes of 2×scr-infected mice. These cells appeared to co-localize with CD206 expressing cells (**Fig 3A**) and Mac2 (**S4 Fig).** In contrast, we detected no ZIKV in the testes obtained from mice infected with the 2×511(T) virus (**Fig 3A**), which looked nearly identical to the testes obtained from mock infected mice. To further quantify these data, we evaluated ZIKV-infected cells among the CD206-positive and -negative populations using flow cytometry. These analyses, shown in **Fig 3B**, revealed that the vast majority of the ZIKV-infected cells within the testes of mice infected with the 2×scr virus were CD206 positive **(Fig 3B**). Likewise, ZIK-infected cells were not detected in mock-inoculated mice or mice infected with the 2×511(T) virus (**Fig 3A and 3B**). Together, these data suggest that CD206/mir-511-3p-expressing cells are the primary early targets for ZIKV replication in the testicular interstitium.

To investigate the possibility that infected M1 macrophages from the periphery may be infiltrating the testicular interstitium, which could be delivering ZIKV to M2 macrophages, we immunophenotyped the myeloid cell populations in the testes of mock-inoculated mice as well as mice infected with either 2×scr or 2×511(T) virus. Using flow cytometry, we identified M1 macrophages (F4/80^+^CD11c^+^CD206^low^) and M2 macrophages (F4/80^+^CD11c^-^CD206^hi^). The M2 macrophages were further differentiated into interstitial (MHCII^-^) or peritubular (MHCII^+^) macrophages as previously described [46]. We found that while there were M1 macrophages clearly identified in mouse testes, there were no significant differences in the number of M1 macrophages at 3dpi between the groups (**Fig 3C**). However, evaluation of M2 macrophages revealed more interstitial M2 macrophages in 2×scr infected testes than in 2×511(T) or mock-inoculated groups, and more peritubular M2 macrophages in the mock- and 2×511(T) group, consistent with the microscopy data (**Fig 3A**). Peritubular and interstitial macrophages both express CD206, while infiltrating M1 macrophages have much lower expression (**Fig 3D**). Collectively, these data show that the majority of ZIKV-infected cells within the testes occur in CD206-positive macrophages, which are enriched during infection.

### Infection of testicular macrophages significantly upregulates the production of macrophage-associated chemokines

**Fig 4:**
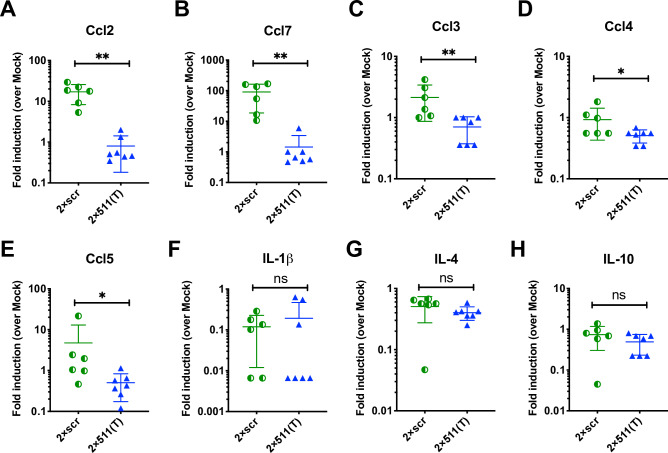
Infection of testicular macrophages is necessary for the production of macrophage-associated inflammatory mediators. Testis homogenates from AG129 mice infected with either 2×scr (n = 6) or 2×511(T) (n = 7) or mock inoculated mice (n = 3) were collected at 3dpi and analyzed for chemokine and cytokine production by multiplexed ELISA. Fold induction over values found in mock-inoculated mice is shown as pg/g of tissue. (**A-E**) macrophage-associated chemokines and (**F-H**) representative markers of inflammation. Significance was determined by a two-tailed Mann-Whitney test. * indicates p<0.05, ** indicates p<0.01.

We next sought to determine the impact of ZIKV infection of these testicular macrophages on the testicular inflammatory response. To do this, we analyzed testes homogenates from AG129 mice infected with 2×scr or 2×511(T) virus for a panel of 8 mouse cytokines and chemokines by multiplexed ELISA. Following infection with the 2×scr virus, we observed the most striking differences for Ccl2 and Ccl7, two macrophage-associated chemokines, with ~10-100-fold increase in the testes on day 3 post infection over testes obtained from mock-inoculated mice. However, this elevation was completely ablated in the testes isolated from mice that were infected with the 2×511(T) virus (**Fig 4A and 4B**). Smaller changes in chemokine production were also observed for several other macrophage associated chemokines, including Ccl3, Ccl4, and Ccl5 (**Fig 4C–4E)**. Other inflammatory cytokines, such as IL1β, IL-4, and IL-10 showed no differences in the testes between the 2×scr- or 2×511(T)-infected mice (**Fig 4F–4H)**. These data suggest that infection of CD206/mir-511-3p -expressing macrophages was required to produce these inflammatory chemokines.

### Soluble factors produced in the presence of ZIKV-infected macrophages disrupt the SCB

**Fig 5:**
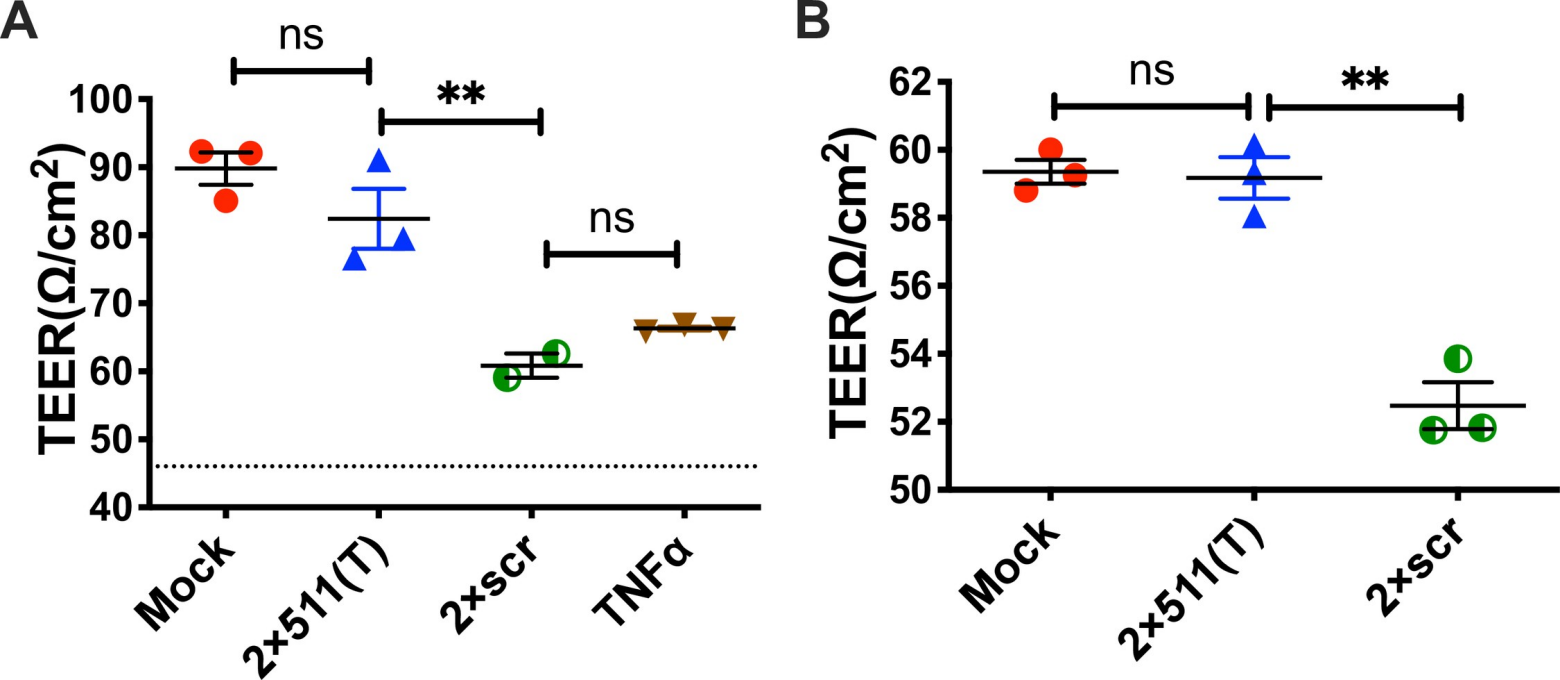
Soluble factors produced in the presence of ZIKV-infected macrophages can disrupt the Sertoli cell barrier. A murine Sertoli cell line, 15-P1, was grown to confluency in transwells. Cells were then exposed to testis homogenates from AG129 mice that were infected with 2×scr (n = 2 for experiment depicted in (**A**), and n = 3 for experiment depicted in panel (**B**)), 2×511(T) (n = 3), or mock inoculated (n = 3). One hundred nanograms of murine TNF⍺ was also included as a positive control (**A**). A voltage was applied across the transwell 24 hours post exposure, and resistance was measured. Transepithelial electrical resistance (TEER) was calculated as ohms/cm^2^. Since 15-P1 monolayers had grown to different peak TEER values in two independent experiments, raw TEER values are reported separately (**A**) and (**B**) for two independent, double blinded experiments. Significance was determined by one way ANOVA. ** indicates p<0.01.

As our data identified CD206-positive cells in the testes as necessary early targets of ZIKV infection for viral dissemination into the seminiferous tubules, we next examined the possibility that the inflammatory milieu in the presence of infected macrophages may assist in viral transport across the SCB, which would then permit the infection of the Sertoli cells within the seminiferous tubules. To do this, we developed an SCB system using the mouse Sertoli cell line, 15-P1. These cells were grown to confluency and allowed time to form tight junctions, which was signified by a stabilization in the measurement of transepithelial electrical resistance (TEER), a measure of monolayer permeability. Barrier functionality was confirmed by measuring GFP-dextran leakage through the monolayer of 15-P1 cells (**S5 Fig**). Filtered homogenates from testes collected from mock-inoculated mice, as well as 2×scr or 2×511(T) infected mice, were added to the upper transwell compartment for 24 hours in a double-blind manner. A voltage was then applied to the monolayer, and resistance was measured across the transwells. TEER was then calculated to evaluate the permeability of the Sertoli cell monolayers. As shown in **Fig 5A**, testes homogenates from mice infected with the 2×scr virus caused a significant decrease in TEER values, that was very similar to disruption of the monolayer caused by TNF⍺, a positive control for barrier disruption. In contrast, exposure of 15-P1 cells to testes homogenates derived from 2×511(T)-infected mice did not significantly alter the permeability of the monolayer and was comparable to the monolayer exposed to testes homogenates from mock-inoculated mice. These findings were consistent across two independent experimental replicates (**Fig 5A and 5B**), suggesting that soluble factors produced only in the presence of ZIKV-infected macrophages are critical for the breakdown of the SCB.

### Targeting multiple regions of ZIKV genome for mir-511-3p inhibits viral replication in peripheral mouse organs and negatively affects viral immunogenicity following low, but not high dose of virus inoculation

**Fig 6:**
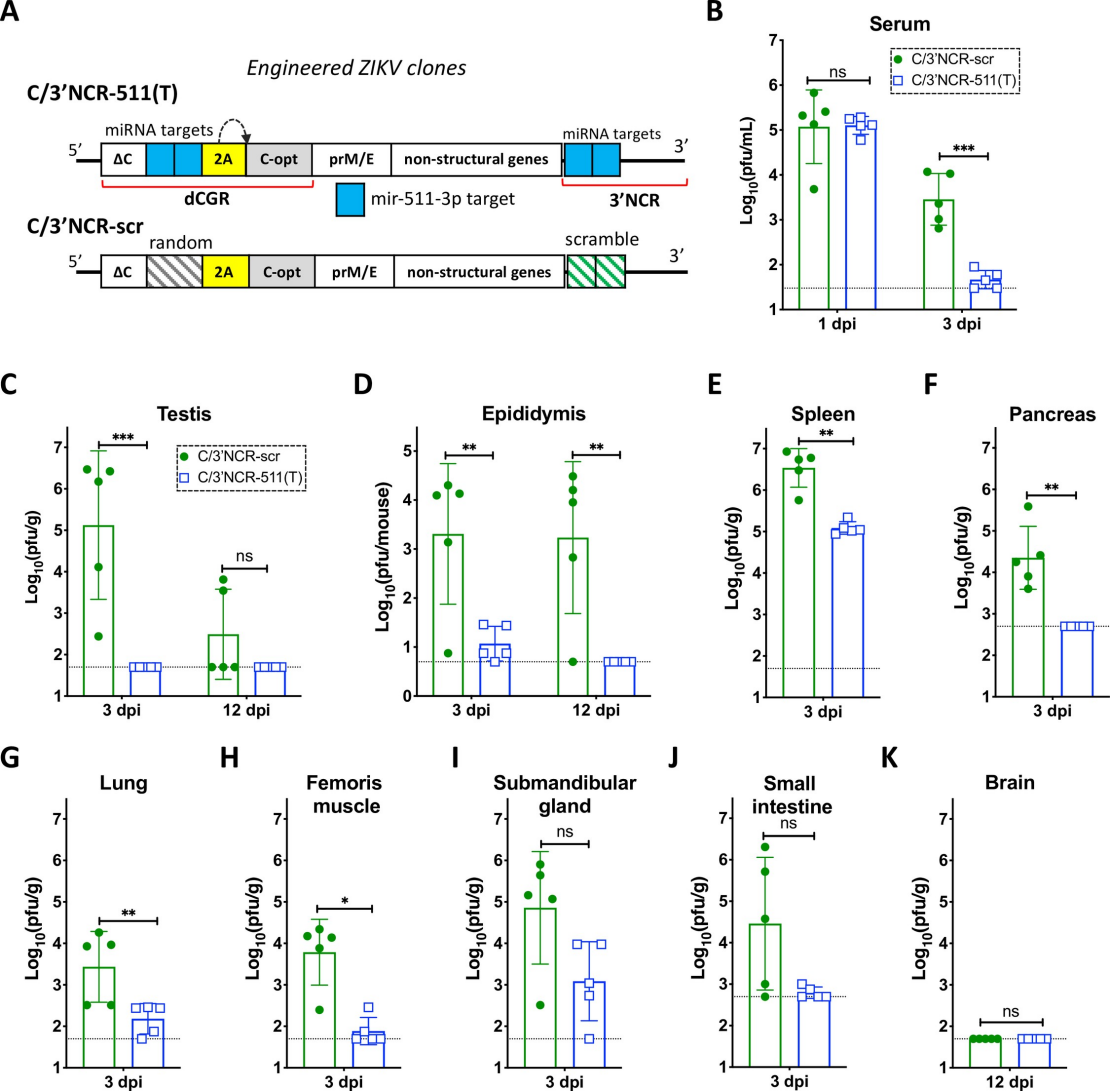
Insertion of multiple copies of mir-511-3p target sequences into distant regions of the ZIKV genome inhibits viral replication in peripheral mouse organs. **(A)** Schematic representation of viruses used in the mouse studies. (**B-K**) Male AG129 mice (5 per group) were infected IP with 10^6^ pfu of C/3’NCR-511(T) or C/3’NCR-scr virus and were bled at 1 dpi and sacrificed at 3 dpi, or were not bled and sacrificed at 12 dpi. (**B)** Mean virus titer ± SD in the serum at 1 and 3 dpi. (**C-D**) Mean virus titer ± SD in the testes (**C**) and epididymis (**D**) at 3 dpi and 12 dpi. (**E-K**) Mean virus titer ± SD in the spleen (**E**), pancreas (**F**), lung (**G**), femoris muscle (**H**), submandibular gland (**I**) and small intestine (**J**) at 3 dpi, and in the brain (**K**) at 12 dpi. The dashed lines indicate the limit of virus detection: in the serum [1.5 log_10_ pfu/mL]; in the testes, spleen, lung, femoris muscle, submandibular gland and brain [1.7 log_10_ pfu/g of tissue]; in the pancreas and small intestine [2.7 log_10_ pfu/g of tissue]; and in the epididymis [0.7 log_10_ pfu/mouse]. Differences between viral titers in the serum, testes and epididymis were compared using two-way ANOVA, and differences between viral titers in other organs were compared using two-tailed Mann-Whitney test. *** p<0.001; **** p*<* 0.0001; ns denotes p>0.05.

Selective restriction of ZIKV replication in M2 macrophages/moDCs by miRNA targeting effectively blocks testicular infection, suggesting that this approach could improve the safety of the live-attenuated vaccine candidate against ZIKV. However, it remains uncertain to which extent ZIKV infection of CD206/mir-511-3p expressing cells is a prerequisite for the efficient development of adaptive immunity by the infected host. To address this, first we modified 2×511(T) to increase miRNA target stability and prevent formation of escape mutants under miRNA-mediated selective pressure (**S3 Fig**). Previously, we showed that insertion of multiple copies of miRNA targets into distant regions of flavivirus genome minimizes the probability of formation of escape mutants, which may be capable of infecting cells expressing these miRNAs [47–49]. Correspondingly, we introduced duplicated capsid gene region (dCGR) into genome of 2×511(T) virus and inserted two additional target sequences for mir-511-3p between two copies of capsid protein gene, generating C/3’NCR-511(T) virus (**Fig 6A** and **S6 Fig**). To account for non-miRNA mediated effects of genome rearrangement on ZIKV replicative fitness and immunogenicity, we used previously described C/3’NCR-scr virus [17], which does not contain functional miRNA targets in either dCGR or the 3’NCR (**Fig 6A**).

Infection of AG129 mice with 10^6^ pfu of C/3’NCR-511(T) or C/3’NCR-scr virus caused similar viremia at 1 dpi, at which point ZIKV titer in the serum reaches its maximum (**Fig 6B**). However, at 3 dpi, the serum titer of the C/3’NCR-511(T) was significantly lower as compared to the titer of C/3’NCR-scr, suggesting accelerated clearance of mir-511-3p targeted virus from the mouse blood, similar to results obtained with the 2x511(T) construct (**Fig 2A**). These data suggest that CD206/mir-511-3p expressing macrophages/moDCs might be an important source of ZIKV in the blood during the late ‘viremic’ phase of the infection (after 1 dpi), but not during early infection or dissemination of the virus from the site of inoculation (prior to 1 dpi). In the testes, C/3’NCR-511(T) was not detected at 3 or 12 dpi (**Fig 6C)**. Moreover, replication of C/3’NCR-511(T) at 3 dpi was also significantly (**Fig 6D–6H;** p<0.05) or moderately (**Fig 6I and 6J;** p>0.05**)** attenuated in several peripheral mouse organs as compared to that of C/3’NCR-scr virus. This indicates that M2 macrophages/moDCs are important targets for visceral ZIKV replication following its hematogenous dissemination from the site of infection. Sequence analysis of the C/3’NCR-511(T) virus recovered from the serum at 1 and 3 dpi (n = 3) and from the spleen (n = 5) or submandibular gland (n = 3) at 3 dpi demonstrated that all miRNA targets remained stable.

Similar to the testis, ZIKV infection of the epididymis follows a biphasic kinetics of replication [17]. During the early phase of infection (3 dpi), ZIKV primarily targets cells located in the epididymal interstitium, followed by infection of the cells that form spermatozoa-containing tubules of epididymis at 9–12 dpi [17]. Compared to the titer of C/3’NCR-scr virus, the titer of C/3’NCR-511(T) was significantly reduced in the epididymis at 3 dpi, and the virus was not detected in the organ at 12 dpi (**Fig 6D**). This implies that ZIKV replication in the mir-511-3p expressing cells of epididymis (as in the cells of the testis, see **Fig 2C**) during the early phase of infection is a prerequisite for progression of viral infection to the late phase. Interestingly, the C/3’NCR-scr virus was not detected in the brain of AG129 mice at 12 dpi but it was found at a moderate titer in the testis of 2 out of the 5 mice at 12 dpi (**Fig 6K**). This indicates that non-miRNA-mediated factor(s) such as formation of dCGR have more profound attenuating effect on ZIKV replication in the CNS as compared to that in the epididymis or the testis, and suggests that susceptibility of mouse brain to the disseminated ZIKV infection (see **Fig 2B**) does not surpass susceptibility of the testis.

**Fig 7:**
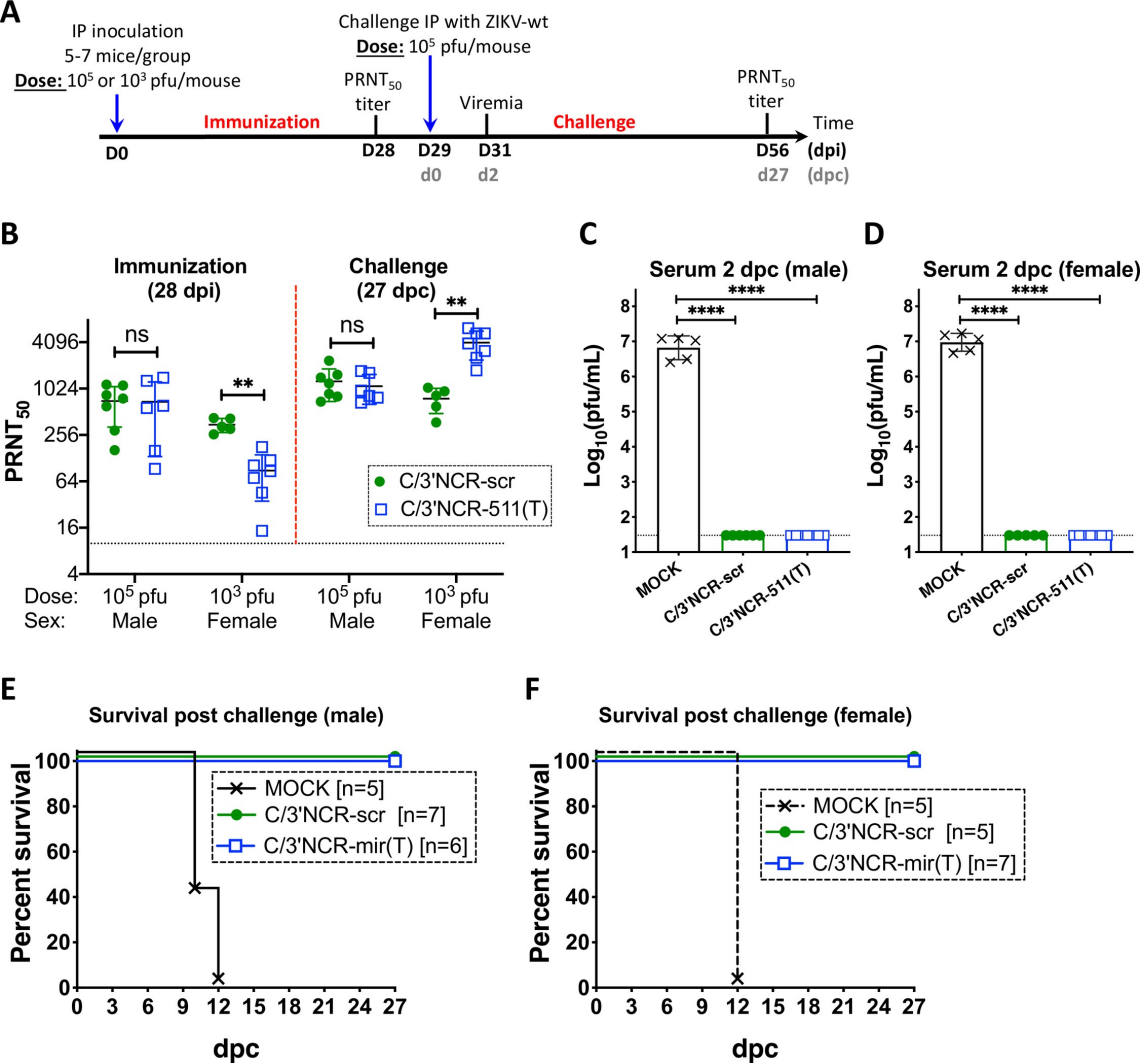
Immunogenicity and protective efficacy of the ZIKV containing multiple copies of mir-511-3p target sequences in AG129 mice. **(A)** Adult male or female AG129 mice were mock inoculated (n = 5) or IP infected with 10^5^ (male) or 10^3^ (female) pfu of C/3’NCR-511(T) or C/3’NCR-scr. On dpi 28 and 56, animals were bled to determine NA titer, which is presented as mean ± SD (**B**). On dpi 29, animals were challenged IP with 10^5^pfu of wt ZIKV (strain Paraiba_01/2015). At 2 days post challenge (dpc) mice were bled to determine viremia (**C, D)** and monitored for survival (**E, F**). The dashed lines indicate the limit of virus detection in the serum [1.5 log_10_ pfu/mL (**C, D**)] or indicate the limit of NA titer detection (**B**). Differences between viral titers in the serum were compared using two-way ANOVA (**C, D**). Differences between NA titers were compared using two-tailed Mann-Whitney test (**B**). ** p<0.01; **** p*<* 0.0001; ns denotes p>0.05. Data for male and female mice is presented as a summary of two and one independent experiments, respectively.

To evaluate the effect of mir-511-3p targeting on ZIKV immunogenicity, we inoculated AG129 mice IP with 10^5^ pfu (male) or 10^3^ pfu (female) of C/3’NCR-511(T), C/3’NCR-scr or diluent alone (mock) and compared ZIKV-specific neutralizing antibody (NA) response after immunization and after a challenge with the parental ZIKV strain (Paraiba_01/2015) (**Fig 7A)**. Immunization with high dose (10^5^ pfu) of both viruses induced comparable NA titers at 28 dpi (**Fig 7B**). In contrast, low dose (10^3^ pfu) immunization with C/3’NCR-511(T) was associated with a significant reduction in NA response as compared to that of C/3’NCR-scr virus (**Fig 7B**). All mice infected with high or low dose of either C/3’NCR-511(T) or C/3’NCR-scr virus did not develop viremia at 2^nd^ day post challenge (dpc) with wt ZIKV (**Fig 7C and 7D**), and all mice remained healthy during 27 days observation period (**Fig 7E and 7F**). In contrast, all mock-inoculated mice (n = 5) developed high titer of wt ZIKV viremia at 2 dpc (**Fig 7C and 7D**) and died within 12 dpc (**Fig 7E and 7F)**. The titer of ZIKV-specific NA in mice inoculated with a high dose of C/3’NCR-511(T) and C/3’NCR-scr remained similar following the challenge with wt ZIKV (**Fig 7B**). In contrast, a substantial (∼40-fold) increase in the ZIKV-specific NA titer was detected in mice immunized with C/3’NCR-511(T), but not with C/3’NCR-scr virus, after the challenge with wt ZIKV (**Fig 7B**). Together, these data indicate that ZIKV infection of the cells expressing mir-511-3p following exposure to low (but not high) dose of inoculation is required for efficient induction of NA response.

## Discussion

In this study, we present evidence implicating testicular macrophages/moDCs as an early target required for successful ZIKV testicular infection. Numerous earlier studies have implicated both macrophages and DCs as the principal host cells for a number of flaviviruses in both lymphoid and non-lymphoid organs ([50–56], reviewed in [57]), including ZIKV ([19, 28], reviewed in [58]). The majority of these studies relied on co-localization approaches, the main weakness of which is the difficulty of establishing causal connection between ability of the virus to infect a particular type of cells and the corresponding pathobiological effect of these infections on the infected host. Attempts also have been made to delineate the role of macrophages in the dissemination of flaviviruses from the site of infection using compounds that selectively deplete this cell population in the host [59–62]. However, results of these studies are difficult to interpret, since depletion of macrophages causes conflicting effects on the dynamics of virus-host interaction. Thus, if macrophages are the true source of viral replication, then depletion of macrophages should reduce a number of potential sites for virus production, which should lead to a reduction in the virus titer in the host and ameliorated pathologies. Alternatively, macrophages are also important sensors and activators of innate and adaptive immune reactions, which are important for the virus clearance. This suggests that a systemic reduction in macrophage abundance could lead to more robust virus replication and aggravated pathologies. As a result, the outcome of macrophage depletion experiments depends on a specific balance between these two opposite factors, of which the immunoinhibitory effects of macrophage depletion usually prevails.

To overcome these limitations, we applied an miRNA targeting approach [17, 63–65] to study ZIKV cellular tropism specifically in the testes. This approach allows for selective restriction of ZIKV replication in the cell type of interest without the systemic depletion of that cell type from the host. The main drawback of this approach is that only a limited number of host miRNAs exhibit expression patterns restricted to a specific cell type. For instance, reliance on mir-511-3p targeting does not allow for precise differentiation between M2 macrophages and moDCs, or the relative contribution of both cell types in ZIKV infection of the testicular interstitium. Macrophages (M2 macrophages in particular) are the most abundant leukocyte population in the mammalian testes [27, 66]. For primates, it was shown that M2 macrophages expressing CD169 marker are ~200 times more abundant compared to all subtypes of testicular DCs [23]. We therefore speculate that the alteration(s) in ZIKV pathobiology, which were observed for mir-511-3p targeted viruses in the testes, are caused primarily by the changes in the capacity for ZIKV to infect M2 macrophages.

Targeting of the ZIKV genome for mir-511-3p did not prevent virus dissemination from the site of infection into the blood (**Fig 2A**), indicating that M2 macrophages/moDCs are not a major source of ZIKV replication/production in AG129 mice at the site of exposure. The peak viremia of the 2×511(T) was similar to that of the 2×scr control virus, which suggests unrestricted dissemination of 2×511(T) virus into all organs, including the testes, by a hematogenous route and regardless of miRNA restriction. For dengue virus, it was shown that in order to develop viremia, and thus a systemic infection, the virus must first infect and replicate in cells of hematopoietic origin at the site of exposure [64]. We have come to a similar conclusion for ZIKV, since targeting of the ZIKV genome for hematopoietic cell specific miRNA mir-142-3p greatly reduces ZIKV viremia (**S7 Fig**), suggesting that early replication of ZIKV (and early viremia) occurs in the cells that express mir-142-3p, but do not express mir-511-3p. We, therefore, speculate that neutrophils or monocytes (but not macrophages) might be the earliest targets for ZIKV replication at the site of infection. Indeed, it has been shown that depletion of neutrophils (but not monocyte-derived macrophages) reduces West Nile virus (WNV) viremia in mice [39], while in humans ZIKV primarily infects monocytes among other types of peripheral blood mononuclear cells [41]. Moreover, both neutrophils and monocytes express high levels of mir-142-3p and do not express mir-511-3p, which is in agreement with the development of viremia for ZIKV clones targeted for mir-511-3p but not for mir-142-3p ((**Fig 2** and **S7 Fig**), also see [34, 38, 67]).

Cells expressing mir-511-3p are not confined to the testes and are dispersed quite homogeneously throughout the organism [38]. It is, therefore, possible that for efficient dissemination, ZIKV might need to infect and replicate in some population(s) of mir-511-3p-expressing cell(s) located outside the testis prior to entering testicular interstitium. Currently, we cannot completely exclude such possibility, considering that clearance of ZIKVs containing targets for mir-511-3p from the mouse blood occurs significantly faster than the clearance of scramble control viruses (**Figs 2A and 6B**). However, we showed that replication in mir-511-3p-expressing cells is not required for ZIKV dissemination into the mouse brain (**Fig 2B**), and cells of the brain do not appear to be more susceptible to the ZIKV infection as compared to the cells of the testis (see **Fig 6C and 6K**). Moreover, by the 3 dpi, the C/3’NCR-511(T) virus was able to disseminate and replicate quite efficiently in the spleen and submandibular gland (**Fig 6E and 6I**), although the titer of the virus in these organs was lower than that of C/3’NCR-511(T). Therefore, it seems unlikely that resistance of the testicular interstitium to the infection with 2×511(T) and C/3’NCR-511(T) viruses is attributed to the inability of these viruses to develop a disseminated infection from the site of inoculation. A more plausible explanation suggests that low viremia of mir-511-3p targeted viruses at 3 dpi is a direct consequence (and not the cause) of the attenuated replication of these viruses in the peripheral organs (especially in the spleen and possibly other lymphoid organs), but not in the brain (**Figs 2 and 6**), and that mir-511-3p expressing cells appear to be one of the most susceptible targets for ZIKV in the majority of these organs. However, the detailed role of splenic macrophages (and macrophages located in other lymphoid organs) as a source of circulating ZIKV virions requires further investigation.

Immunophenotyping of the testes revealed that there are no significant differences in the total number of M1 macrophages in the testes of mice that received the 2×scr virus compared to mice that received the 2×511(T) virus, indicating that increased infiltration of M1 macrophages is not responsible for the infection in the testes. Interestingly, 2×scr infected testes have a marked increase in the number of interstitial macrophages compared to the 2×511(T) virus, with the inverse being true of peritubular macrophages (**Fig 3C**). While both populations express CD206 (**Fig 3D**), infection remains interstitial (**Fig 3A**). We believe that this is due to the recruitment of M2 macrophages and/or the polarization of bone marrow derived macrophages into the M2 type. Indeed, these macrophages are known to be attracted to Ccl7 and Ccl2 which are known to drive differentiation of bone marrow derived, unpolarized macrophages into M2 macrophages [68, 69]. Both of these were the highest expressed chemokines that we identified in the homogenates of testes infected with 2×scr, but not 2×511(T) or mock inoculated testes **(Fig 4)**. Taken together, these data suggest a potential mechanism by which early infection of M2 macrophages in the testes leads to the recruitment of unpolarized macrophages and their polarization into the M2 type, thus providing new targets for infection in the testicular interstitium.

Restriction of ZIKV replication in M2 macrophages/moDCs by mir-511-3p targeting caused almost complete prevention of ZIKV accumulation in the testes at 3 dpi (**Fig 2C**), implicating these cells as primary early ZIKV targets in this organ. At this time point, the control 2×scr virus was primarily found in the cells located in the testicular interstitium (**Figs 3 and S2)**. Approximately 95% of 2×scr-infected cells within the interstitium stained positive for CD206 by flow cytometry (**Fig 3B)**, indicating that the majority of the ZIKV-infected population at this time are of the M2 phenotype. Thus, ablation of the capacity for ZIKV to replicate in this compartment also restricts the virus from replication in the interstitium in general. This indicates that testicular macrophages are critical targets for early viral propagation in the testes.

Prior to this study, it remained unclear whether infection of the testicular interstitium was a necessary step for downstream infection of the Sertoli cells and developing germ cells in the seminiferous tubules during late phase of testicular infection, or if these infectious events occur independently of one another. Previously, we showed that late phase of testicular infection (12 dpi) can be blocked by the insertion of targets for miRNAs that are selectively expressed only in the seminiferous tubules [17], while these targets have no effect on initial stage of ZIKV replication (3 dpi) in the testicular interstitium. Here, we observed that targeting of ZIKV genome for mir-511-3p prevents virus accumulation during both early and late phases of infection (**Fig 3C**), linking early and late ZIKV infection in the testes into a single pathobiological process, and suggesting that early ZIKV replication in the testicular interstitium is a prerequisite for the progression of virus infection into seminiferous tubules.

It is not precisely clear how ZIKV from the interstitium is capable of infecting cells located inside the seminiferous tubules, which is an immune-privileged site. In mammals, seminiferous tubules are separated from interstitium by a layer of basal lamina and tight junctions formed by Sertoli cells (reviewed in [70]), known as the SCB. Previously, Siemann et al. provided evidence suggesting that *ex vivo* ZIKV infected macrophages secrete inflammatory mediators, which compromises the integrity of an artificial SCB grown on a transwell [22]. Our study supports and extends these findings using testis homogenates from ZIKV-infected mice that received the 2×scr virus, which were capable of disrupting the SCB, while the homogenates from mice infected with the 2×511(T) did not (**Fig 5**). While it is not clear if this disruption is a direct result of chemokine production or some other factor produced in response to ZIKV infection, it is clear that this factor is soluble and only present following infection of testicular macrophages. It is possible that this phenotype is being driven by the production of NS1 (which should be similar to the mechanism that was proposed for ZIKV-induced placenta permeability [71]), or by chemokine production (in accordance with [22]), or by virus itself, or by some other soluble factor produced in response to viral infection. Future work is needed to characterize the mechanism by which this interstitial infection can result in the downregulation and/or disruption of the tight junctions of the SCB.

Our data shows that ZIKV infection of mir-511-3p expressing cells is not required for induction of anti-ZIKV protective immunity, as all AG129 mice inoculated with high or low dose of C/3’NCR-511(T) virus were protected against the challenge with wt ZIKV (**Fig 7C–7F**). This suggests that mir-511-3p targets could be a useful tool for controlling replication of live-attenuated ZIKV vaccine candidate virus(es) in the peripheral organs (i.e. testis or epididymis). However, a considerable reduction of the ZIKV-specific NA response was observed following exposure of mice to a low, but not the high, dose of immunization with C/3’NCR-511(T) virus (**Fig 7B**). This suggests that comprehensive dose-response study will be required as part of safety and efficacy evaluations of prospective live-attenuated ZIKV vaccine viruses that contain targets for mir-511-3p. It seems unlikely that reduction of the NA response following C/3’NCR-511(T) inoculation can be attributed to the sex-related differences in immune system activation (we used male mice for high dose of inoculation and females for low dose of C/3’NCR-511(T)). For instance, NA response was similar in mice exposed to both doses of a control C/3’NCR-scr virus (**Fig 7B**). More plausible explanation for the observed differences in viral immunogenicity suggests that insufficient number of antigen presenting cells (which would be necessary for induction of a strong NA response) are getting infected with C/3’NCR-511(T) following exposure to a low dose of the viruses. Since C/3’NCR-scr virus (but not the C/3’NCR-511(T)) can efficiently replicate in the mir-511-3p-expressing cells of many peripheral organs (**Fig 6**), it is likely that these ‘secondary infections’ compensate for the insufficient number of ‘primary infections’ of the antigen presenting cells, providing a boost to the immunogenicity of the C/3’NCR-scr, but not the C/3’NCR-511(T) virus.

Replication and immunogenic properties of the ZIKV virus carrying multiple targets for mir-511-3p in some respects resemble behavior of single replication cycle flavivirus particles [72]. Such particles cannot infect cells located in the peripheral mouse organs, while inducing strong anti-viral NA responses following inoculation of mice and non-human primates with a high (but not with a low) dose of virus particles [73, 74]. Interestingly, using mice treated with macrophage depleting agent, Winkelmann *et* al. showed that subcapsular sinus macrophages are not required for T cell activation following inoculation with high dose (10^7^ infectious units) of WNV particles [60], which is consistent with induction of strong NA responses following inoculation of AG129 mice with high dose of C/3’NCR-511(T) virus. Importantly, the same study [60] showed that depletion of macrophages does not inhibit, but rather promotes dissemination of the WNV-infected cells from the site of incoulation. This is consistent with replication profle of ZIKV carrying targets for mir-511-3p in AG129 mice, supporting a suggestion that initial dissemination of ZIKV into peripheral organs (testis) can occur without M2 macrophages/moDCs involvement.

Unlike in humans, in mice ZIKV cannot antagonize IFN responses and disseminate from the site of inoculation [75]. We, therefore, relied on immunocompromised AG129 mouse model to study ZIKV tropism in the testis. This and other similar mouse models of ZIKV pathogenesis [42] employed transient or permanent aberration(s) in IFN signaling pathway to achieve efficient virus dissemination from site of inoculation. However, it is possible that such abnormalities can artificially increase sensitivity of various cell populations (including mir-511-3p expressing macrophages and moDCs) to ZIKV infection. This would cause overestimation/misrepresentation of the role of these cells in ZIKV pathogenesis. Therefore, it will be important to independently validate the role of CD206 expressing cells in ZIKV pathogenesis using immunocompetent non-human primate model of infection. Alternatively, targets for miRNA mir-511-3p may be introduced into recently developed mouse-adapted Dak-41525 strain of ZIKV, followed by evaluation of resulted viruses in immunocompetent hSTAT2 KI mice [76]. Finally, taking into account that CD206 has been implicated as a binding receptor of all four dengue virus serotypes on human macrophages [52], it also will be interesting to elucidate potential involvement of CD206 in the cell entry of ZIKV.

### Conclusions

We have demonstrated that miRNA targeting of ZIKV is an effective tool that can be used for understanding the role of specific cell types in the progression of infection, in a tissue dependent manner. We have identified M2 macrophage/moDC cells expressing CD206 and miRNA mir-511-3p as critical targets for ZIKV in the testicular interstitium. Infection of these cells is a necessary step that allows subsequent progression of ZIKV infection to the seminiferous tubules. Our findings provide direct experimental evidence supporting the role of infected testicular macrophages in the disruption of the SCB during ZIKV infection, a hypothesis that was put forward earlier by other group [22]. Finally, we propose that targeting of viral genome for M2 macrophage/moDC cell-specific miRNA can improve the safety profile of the ZIKV live attenuated vaccine candidate.

## Materials and methods

### Ethics statement

All experimental protocols were approved by the NIH Institutional Biosafety Committee. All animal study protocols (ASP) (specifically ASP LID 23E (authorized 07/01/2018) and ASP LID 25E (authorized 12/01/2018)) were approved by the NIAID/NIH Institutional Animal Care and Use Committee (IACUC) and performed in compliance with the guidelines of the NIAID/NIH IACUC. The NIAID Division of Intramural Research (DIR) Animal Care and Use Program, as part of the NIH Intramural Research Program (IRP), complies with all applicable provisions of the Animal Welfare Act (http://www.aphis.usda.gov/animal_welfare/downloads/awa/awa.pdf) and other Federal statutes and regulations relating to animals. The NIAID DIR Animal Care and Use Program is guided by the "U.S. Government Principles for the Utilization and Care of Vertebrate Animals Used in Testing, Research, and Training" (http://oacu.od.nih.gov/regs/USGovtPrncpl.htm). The NIAID DIR Animal Care and Use Program acknowledges and accepts responsibility for the care and use of animals involved in activities covered by the NIH IRP’s PHS Assurance #D16-00602, last issued 6/10/2019. The NIAID DIR Animal Care and Use Program has established and will maintain a program for activities involving animals in accordance with the most recent (2011, 8^th^ edition) of “The Guide for the Care and Use of Laboratory Animals” (ILAR, NRC) (http://oacu.od.nih.gov/regs/guide/guide_2011.pdf). The policies, procedures and guidelines for the NIH IRP are explicitly detailed in NIH Policy Manual 3040–2, “Animal Care and Use in the Intramural Program” (PM 3040–2) and the NIH Animal Research Advisory Committee Guidelines (ARAC Guidelines). Those documents are posted on the NIH Office of Animal Care and Use public website at: http://oacu.od.nih.gov.

### Plasmids and viruses

Sequences for all plasmids are available from the authors upon request. All recombinant infectious cDNA clones of ZIKV were constructed based on ZIKV strain Paraiba_01/2015, isolated during the 2015 epidemic in Brazil. Clones encoding ZIKV-NS3m, 2×scr, and C/3’NCR-scr were described earlier [17, 18]. Sequences that were complementary to mouse mir-511-3p were used to replace both scr sequences in the 2×scr, generating clone 2×511(T) (**S1 Fig**). To generate C/3’NCR-511(T), we replaced random sequence in the dCGR of C/3’NCR-scr (see **Fig 6A** and [17]) with targets for mir-511-3p (**S6 Fig**). The resultant plasmid was subsequently modified by replacing both scr sequences in the 3’NCR with those of mir-511-3p targets (**S6 Fig**).

### Virus recovery from cDNA clones and titration

Viruses were recovered from cDNA clones as described previously [17, 18]. Briefly, 2.5 μg of plasmid DNA was transfected into Vero cells seeded in a 12.5-cm^2^ flask in duplicates using Lipofectamine 2000 reagent. Vero cells were obtained from World Health Organization and used from passage 143 till passage 150. Following DNA transfection, Vero cells were maintained in 5 mL of DMEM (Gibco) supplemented with 10% FBS (HyClone) and 1x penicillin-streptomycin-glutamine solution (Invitrogen). Aliquots of cells supernatant were collected daily and kept at -80°C for virus titer determination. Virus stocks, which were used in all experiments, were made on 3 dpt by supplying clarified Vero cell supernatants with 1x SPG solution (218 mM sucrose, 6 mM L-glutamic acid, 3.8 mM KH_2_PO_4_, 7.2 mM K_2_HPO_4_, pH 7.2) and stored at -80°C. Differences in virus growth kinetics after cDNA transfection were compared using 2-way ANOVA analysis implemented in Prism 8 software (GraphPad Software, San Diego, CA).

Titers of all ZIKV constructs were determined by plaque assay in Vero cells as described previously [18]. Plaques were visualized at 5 dpi by staining Vero cell monolayers in 24-well plates with 0.5% crystal violet solution.

### Growth of miRNA-targeted ZIKV clones in adult AG129 mice and evaluation of virus titer in organs and serum

A colony of type I and type II IFN receptor gene knockout AG129 mice, established from a breeding pair purchased from Marshall BioResources, was maintained at the NIAID/NIH animal facility. Adult (4-6-week-old) male mice were injected IP with 10^6^ pfu (0.1 mL/mouse) of 2×scr, 2×511(T), C/3’NCR-511(T) or C/3’NCR-scr virus diluted in L-15 media (Gibco), which was supplemented with 1x SPG solution (L15/SGP). Mice were bled at 1, 3 and 6 dpi from the submandibular vein for serum collection, and were euthanized at 3, 6 and 12 dpi for organ dissection. Mouse organs and serum samples designated for determination of virus load were stored at -80°C. To determine virus titers in the testis, brain, spleen, submandibular gland, lung, pancreas, small intestine, and femoris muscle, mouse organs were weighted and triturated in 9 volumes of L-15/1xSPG solution. Since various amount of fat tissue remains attached to epididymis during dissection, pair of epididymides from each mouse was not weighted, but directly triturated in 1 mL of L-15/1xSPG solution. Serum and organ homogenates were titrated by plaque assays in Vero cells as described earlier [18, 77].

### Study of immunogenicity in adult AG129 mice and evaluation of neutralizing antibody titer in mouse serum

Immunogenicity of ZIKV clones in adult AG129 was evaluated as described previously [17]. ZIKV clones were evaluated in 4-6-week-old male or female AG129 mice. Mice were infected IP with 10^5^ pfu (male) or 10^3^ pfu (female) of recombinant viruses. Mock inoculated mice received 0.1 mL of L-15 medium supplemented with 1x SPG solution (L15/SPG). At 29 dpi, AG129 mice were challenged IP with 10^5^ pfu of wt ZIKV (strain Paraiba_01/2015). Mice were bled at indicated intervals post infection (**Fig 7A**) to determine viremia and NA titers in the serum against Paraiba_01/2015 strain of ZIKV, using the 50% plaque reduction neutralization assay [78]. Differences between NA titers in the serum were compared using two-tailed Mann-Whitney test implemented in Prism 8 software.

### ZIKV RNA detection by in situ hybridization and immunofluorescence co-staining

Adult (4-6-week-old) male mice were injected IP with 10^6^ pfu of 2×scr or 2×511(T) virus. At 3 dpi, tissues from one testicle were fixed in 10% neutral buffered formalin for 24 hours and placed back into PBS until paraffin embedding. *In situ* hybridization using RNAscope was performed on 8 μm paraffin-embedded sections. Deparaffinization was performed by baking slides at 55°C for twenty minutes. Following this step, slides were washed twice with xylene for five minutes each, twice in 100% ethanol for two minutes each, and slides were dried for five minutes at 60°C. Slides were then incubated with hydrogen peroxide for 10 minutes at RT, and were subsequently washed in diH_2_O. Target retrieval was performed by incubating slides in Target Retrieval solution at 100°C for 15 minutes. Lastly, slides were washed with water and transferred into 100% ethanol for three minutes, then were allowed to dry. Sections were treated with RNAscope Protease Plus and incubated at 40°C for 30 minutes. Slides were then washed with diH_2_O. Fluorescence *in situ* hybridization was subsequently performed according to the manufacturer’s protocol (ACD# 323110) with RNAscope Probe V-ZIKVsph2015 (ACD #467871; binds sense RNA) as previously described [79]. Following *in situ* hybridization, slides were washed twice for five minutes in Tris-buffered Solution containing 0.01% Tween-20 (TBST) and subsequently blocked in a solution of 2.5% goat serum and 2.5% BSA in TBST for 30 minutes at RT. For co-stains, sections were incubated for 1 hour at 4°C with either polyclonal rabbit anti-mannose receptor (CD206, 1ug/mL, Abcam) or monoclonal rat anti-MAC2 antibody (1:500, clone M3/38, Cedarlane) and then detected with either anti-rabbit IgG phycoerythrin or anti-rat IgG phycoerythrin, respectively. Slides were then mounted with Vectashield hard-set mounting medium with DAPI (Vector Laboratories). Slides were analyzed using an AxioImager Z2 microscope (Zeiss) and Zen 2012 software (Zeiss).

### Quantification of CD206 infected cells and immunophenotyping

Adult (4-6-week-old) male mice were injected IP with 10^6^ pfu of 2×scr or 2×511(T) virus or were mock infected. Mice were euthanatized at 3 dpi. Testes were dissociated into single cell suspensions by incubation with 1.25μg/mL collagenase IV and 10 IU DNAse I for one hour, at 37°C shaking. Cells were then washed with PBS, and 2x10^6^ cells were plated in a 96 well plate, in duplicate. Remaining cells were fixed with 4% PFA for 30 minutes at RT.

For CD206 staining, cells were fixed with 4% PFA for 30 minutes at RT. Cells were then permeabilized with BD Cytofix/Perm buffer for 30 minutes at 4°C. Cells were then stained with anti-flavivirus antibody clone 4G2 (1μg/mL) for 1 hour at RT. Cells were washed 3 times and stained with a PE-conjugated goat anti-mouse IgG (1μg/mL) for one hour. Cells were washed 3 times and were then co-incubated with a CD206 antibody. Cells were analyzed on a FACSCaliber. All data analysis was conducted in Flowjo 10.6.1.

For immunophenotyping of macrophages in testes, single cell suspensions were stained for 10 minutes with Zombie Red Live/Dead viability dye. Following washing with PBS, cells were subsequently Fc blocked (TruStain FcX, Biolegend) and stained using the following mouse-specific antibodies: CD45 (clone 30-F11), F4/80 (Clone BM8), CD206 (clone Co688C2), CD11c (clone N418), MHC Class II (clone M5/114.1.2), and CD11b (clone M1/70) for 30 minutes at 4°C in PBS supplemented with 1%BSA and 1mM EDTA. All antibodies were purchased from Biolegend, with the exception of MHC Class II (eBiosciences) and CD11b (BD Biosciences). Cells were subsequently fixed with CytoFix for 30 minutes and were analyzed on a Cytek Aurora cytometer. All data analysis was conducted in Flowjo 10.6.1.

### Cytokine and chemokine protein quantification

Adult (4-6-week-old) male mice were injected IP with 10^6^ pfu of 2×scr or 2×511(T) virus. Mice were euthanatized at 3 dpi. Testes were dissected and 10% organs homogenates were made in PBS and stored at -80°C. Tissue homogenates were evaluated for 8 cytokines/chemokines by multiplex ELISA for the following analytes: Ccl2/MCP-1, Ccl7/MCP-3, Ccl3/MIP-1α, Ccl4/MIP-1β, Ccl5/RANTES, IL1β, IL-4 and IL-10 as previously described [80]. Samples were analyzed on a Luminex MAGPIX platform. For each bead region, >50 beads were collected per cytokine. The median fluorescence intensity of these beads was recorded and used for analysis with the Milliplex Analyst software using a 5P regression algorithm. Expression of these chemokines was measured in pg chemokine/g tissue, as determined using a standard curve of recombinant chemokine. Expression was then normalized to the expression in mock infected mouse testes homogenates. Statistical significance was determined by a two-tailed Mann-Whitney test.

### In vitro SCB model

Murine Sertoli cells 15-P1 (ATCC CRL-2618) were grown on a 3.0 μm pore size transwell (Falcon 353091) in a total volume of 1 mL of DMEM containing 10% FBS, Pen/Strep and L-Glutamine to the density of 5x10^5^ cells/transwell. TEER (Ω/cm2), normalized to a transwell without 15-P1 cells, was measured with an EVOM every 24 hours after plating. When TEER values stabilized, monolayers were washed once with PBS, and then exposed to a 1:10 dilution of testes homogenates in complete DMEM, or 100 ng of murine TNFα as a positive control. TEER was then measured 24 hours post exposure, and TEER values were normalized to values collected in the absence of homogenate (media alone). All TEER experiments were conducted in a double blinded manner. Barrier function was confirmed by evaluating GFP-dextran leakage (50kD) after 2 hours of incubation.

## Supporting information

S1 Fig Genomes of 2×scr and 2×511(T) ZIKV clones. **(A)** Insertion of the scr or mir-511-3p target sequences into genome of ZIKV-NS3m virus. (**B**) Annotated sequences of the 5’ terminus of the 3’NCR for ZIKV-NS3m, 2×scr or 2×511(T) virus. Red arrows and underlined sequences highlight positions of the insertions within 3’NCR. NsiI–a restriction endonuclease cleavage site that was used for construction of the plasmids.
(PDF)

S2 Fig Progression of ZIKV infection into the cells located inside seminiferous tubules of the testes. Adult (4-6-week old) AG129 mice were infected IP with 10^6^ pfu of 2×scr ZIKV. Mice were sacrificed at 3 dpi (**A**) and 12 dpi (**B**) and stained for ZIKV antigen using an anti ZIKV NS2B antibody (GTX133308, GeneTex). Representative images showing the distribution of ZIKV antigen in mouse testes at 3 dpi (**A;** n = 4 mice) or 12 dpi (**B**; n = 6 mice). Blue arrows highlight antigen in the testicular interstitium; red arrows highlight ZIKV antigen in the seminiferous tubules. Scale bars equal 1mm (left panels) and 50μm (right panels). The organs were collected and processed as part of the study, which was reported earlier [17].
(PDF)

S3 Fig Sequencing of 2×511(T) virus isolated from the serum, testis and brain of mouse #5. Adult AG129 mice (n = 7) were infected IP with 10^6^ pfu of 2×511(T). Mice were bled at 1 dpi and sacrificed on 12 dpi. **On the top**—annotated sequence of the 5’ terminus of the 3’NCR for 2×511(T) virus. **On the bottom**–sequencing electrophoregrams of 2×511(T) virus genome isolated from the serum, brain and testes of mouse #5. Red arrows highlight the 3’-end of deleted sequence identified in 2×511(T) virus isolated from the testicular sample. Strikethrough sequence identifies 40 nt deletion in the testicular sample.
(PDF)

S4 Fig Co-localization of Mac2 and ZIKV RNA in mouse testes. Adult AG129 male mice were mock-inoculated (n = 1) or infected IP with 10^6^ pfu of 2×scr (n = 2) or 2×511(T) virus (n = 2). Mice were sacrificed at 3 dpi, and testes were harvested. Testes sections were stained for ZIKV RNA (Red) by *in-situ* hybridization and Mac2 antibody (green) by immunofluorescence co-staining. Scale bars represent 100μm.
(TIF)

S5 Fig Validation of the 15-P1 barrier model. To confirm that the 15-P1 cell line could form a functional barrier, the monolayers were exposed to a GFP-Dextran after TEER values stabilized. The fluorescent intensity was measured from the bottom of the transwells after 2 hours of incubation and compared to a no-cell control and to media alone. Only the no-cell control transwell had GFP-Dextran move to the bottom of the transwell, indicating that the transwells had formed a functional barrier.
(PDF)

S6 Fig C/3’NCR-511(T) virus. **(A)** Schematic representation of C/3’NCR-511(T) virus genome. dCGR–duplicated capsid gene region. C-trn(50AA)–truncated C gene encoding 50 amino acids, which are *cis*-acting elements involved in the virus replication. *—open reading frame (ORF) shifting mutation [Fr Sh (+1)] denotes insertion one nucleotide in the C-trn. C-opt is a full-length copy of C gene, which is responsible for virion assembly. It contains synonymous mutations introduced in each AA codon (except ATG and TGG). 2A - autoprotease 2A from foot-and-mouth disease virus; the curved arrow indicates position of 2A protease cleavage site. Blue boxes indicate targets for mir-511-3p. (**B)** The annotated sequence of the dCGR. (**C**) The annotated sequence of the 3’NCR of C/3’NCR-511(T) virus. XhoI, KpnI and NsiI—restriction endonuclease cleavage sites that were used for cloning of the C/3’NCR-511(T) plasmid.
(PDF)

S7 Fig Targeting of ZIKV genome for hematopoietic cell-specific miRNA mir-142-3p inhibits viremia in AG129 mice. (**A**) Insertion of the mir-142-3p target sequences (highlighted in purple) into ZIKV-NS3m genome generating 2×142(T) virus. (**B**) Expression profile of the mir-142-3p in the selected cells and organs of mice. The graph was constructed based on deep sequencing data of the mouse miRNAs, which was reported earlier [38]. The expression profile is presented as a proportion of the number of reads for mir-142-3p in the cell/organs to the number or reads for this miRNA in the neutrophils, which has the highest expression level among all cell types. HSCs—hematopoietic stem cells, MΦs-macrophages, NK—natural killer cells, MEFs–mouse embryonic fibroblasts. **C—**Growth of 2×scr, 2×511(T) and 2×142(T) viruses in Vero cells after plasmid DNA transfection. **D—**Mean virus titer ± SD in the serum at 1 dpi. AG129 mice (n = 3–6 per group) were infected IP with 10^6^ pfu of 2×scr or miRNA targeted viruses and bled at 1dpi [see **Fig 2** for experiment details]. Differences between the virus titers were compared using one-way ANOVA (**** p < 0.0001; ns—denotes not significant).
(PDF)
