# Supplementary material for: Zika virus tropism during early infection of the testicular interstitium and its role in viral pathogenesis in the testes
Source: PLoS Pathog. 2020 Jul 2;16(7):e1008601. doi: 10.1371/journal.ppat.1008601 (PMC7331987; doi:10.1371/journal.ppat.1008601)
Supplement: S1 Fig — (A) Insertion of the scr or mir-511-3p target sequences into genome of ZIKV-NS3m virus. (B) Annotated sequences of the 5’ terminus of the 3’NCR for ZIKV-NS3m, 2×scr or 2×511(T) virus. Red arrows and underlined sequences highlight positions of the insertions within 3’NCR. NsiI–a restriction endonuclease cleavage site that was used for construction of the plasmids. (PDF) [file ppat.1008601.s001.pdf]

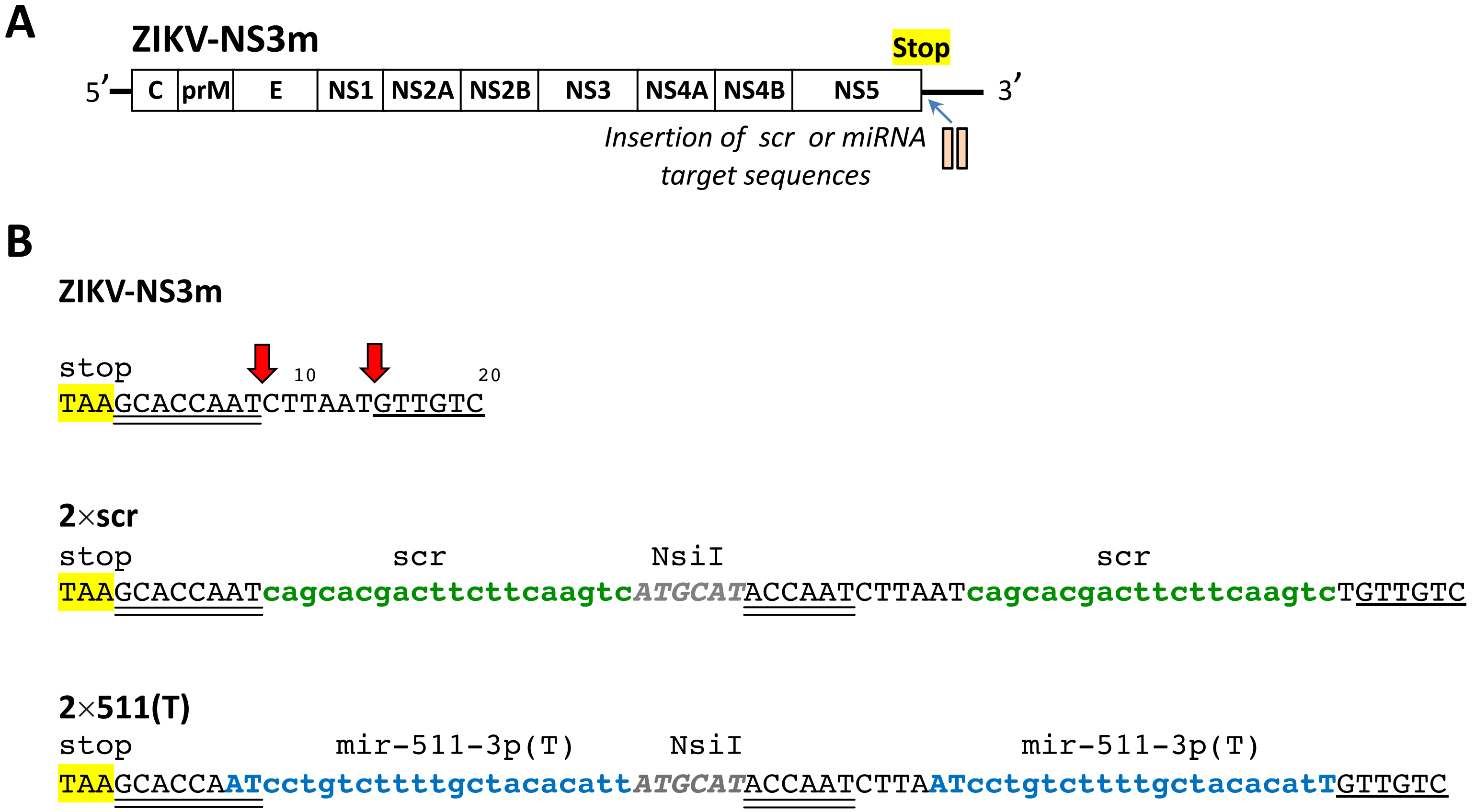
A
ZIKV-NS3m
Stop
5'
C
prM
E
NS1
NS2A
NS2B
NS3
NS4A
NS4B
NS5
3'
Insertion of scr or miRNA target sequences
B
ZIKV-NS3m
stop
10
20
TAAGCACCAATCTTAATGTTGTC
2×scr
stop
scr
NsiI
scr
TAAGCACCAATcagcacgacttcttcaagtcATGCATACCAATCTTAATcagcacgacttcttcaagtcTGTTGTC
2×511(T)
stop
mir-511-3p(T)
NsiI
mir-511-3p(T)
TAAGCACCAATcctgtcttttgctacacattATGCATACCAATCTTAATcctgtcttttgctacacatTGTTGTC
