# Supplementary figures and images for: Zika virus tropism during early infection of the testicular interstitium and its role in viral pathogenesis in the testes
Source: PLoS Pathog. 2020 Jul 2;16(7):e1008601. doi: 10.1371/journal.ppat.1008601 (PMC7331987; doi:10.1371/journal.ppat.1008601)

**A****2×scr; 3 dpi**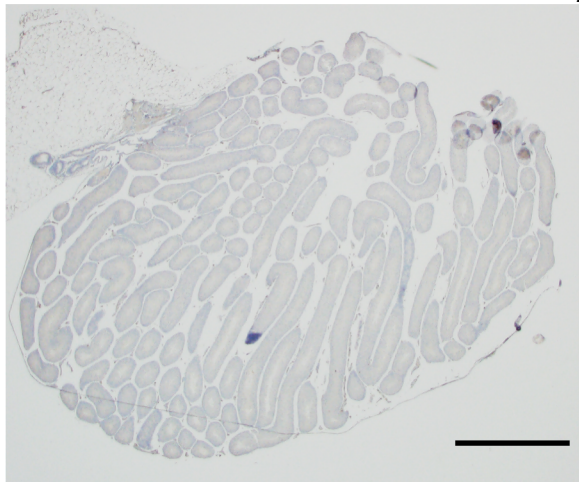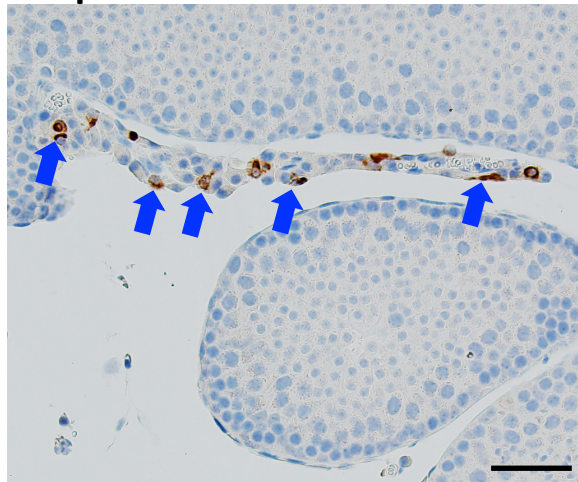**B****2×scr; 12 dpi**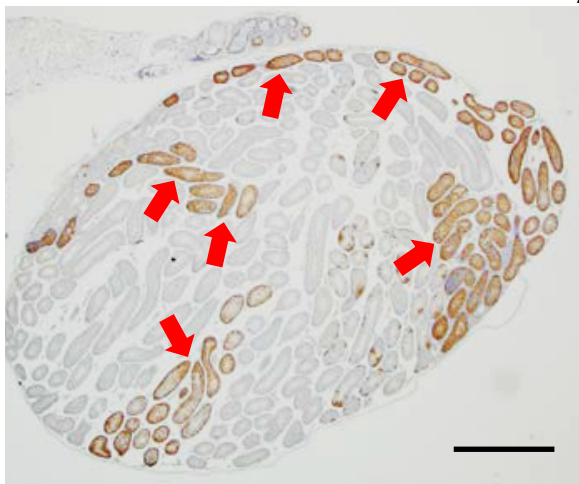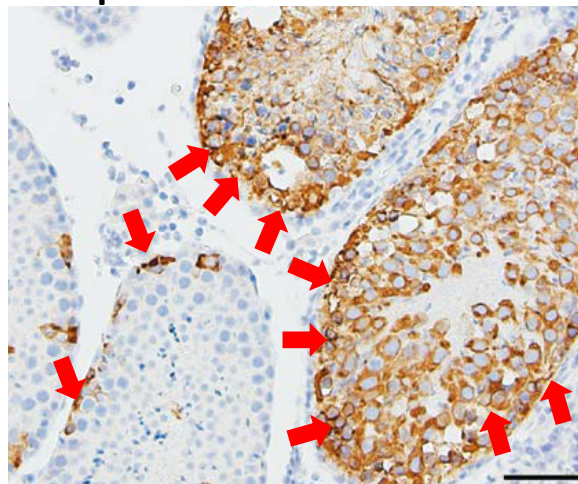

Supplement: S2 Fig — Adult (4-6-week old) AG129 mice were infected IP with 106 pfu of 2×scr ZIKV. Mice were sacrificed at 3 dpi (A) and 12 dpi (B) and stained for ZIKV antigen using an anti ZIKV NS2B antibody (GTX133308, GeneTex). Representative images showing the distribution of ZIKV antigen in mouse testes at 3 dpi (A; n = 4 mice) or 12 dpi (B; n = 6 mice). Blue arrows highlight antigen in the testicular interstitium; red arrows highlight ZIKV antigen in the seminiferous tubules. Scale bars equal 1mm (left panels) and 50μm (right panels). The organs were collected and processed as part of the study, which was reported earlier [17]. (PDF) [file ppat.1008601.s002.pdf]

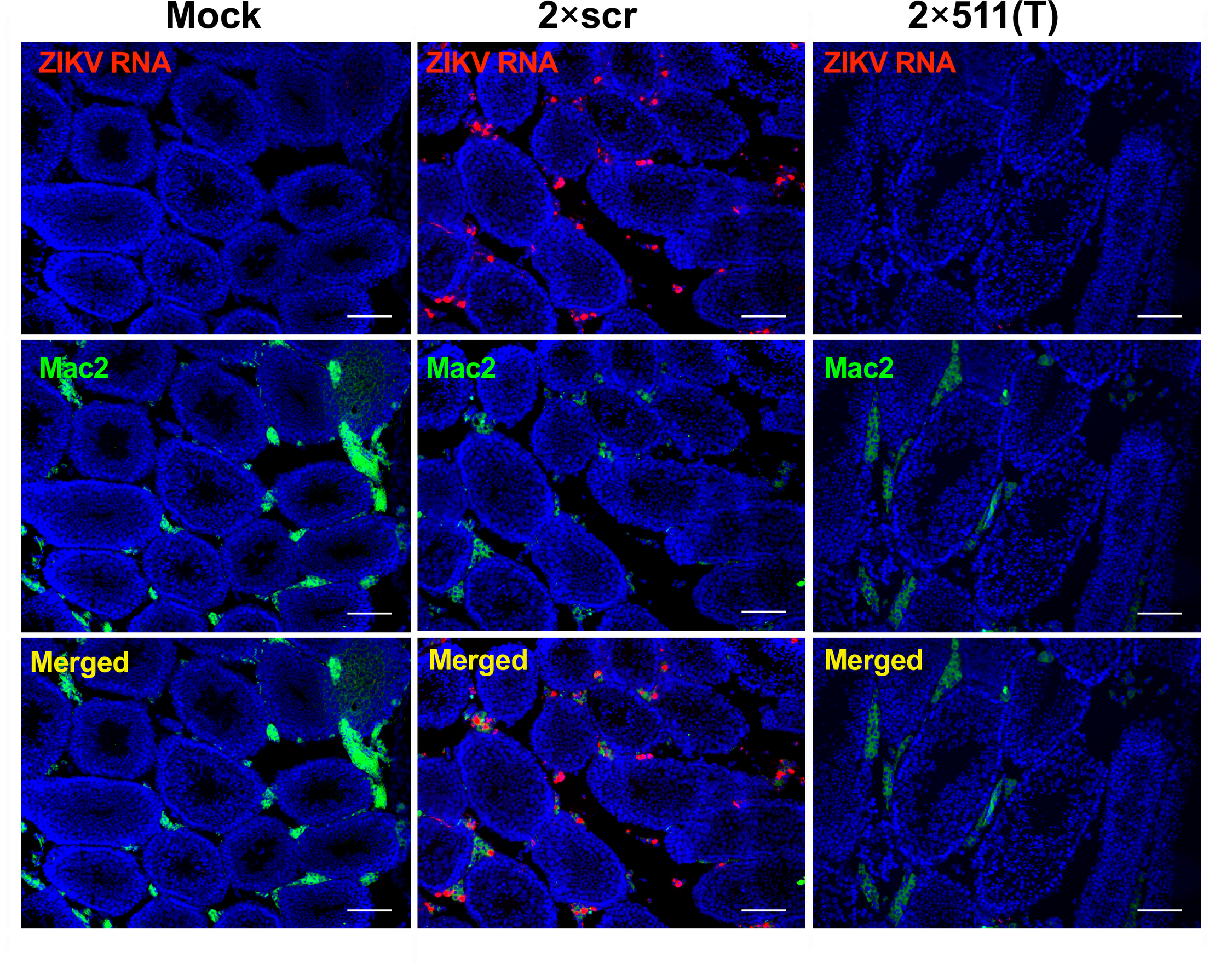

Supplement: S4 Fig — Adult AG129 male mice were mock-inoculated (n = 1) or infected IP with 106 pfu of 2×scr (n = 2) or 2×511(T) virus (n = 2). Mice were sacrificed at 3 dpi, and testes were harvested. Testes sections were stained for ZIKV RNA (Red) by in-situ hybridization and Mac2 antibody (green) by immunofluorescence co-staining. Scale bars represent 100μm. (TIF) [file ppat.1008601.s004.tif]

# 15P1 Cells form a Functional Barrier

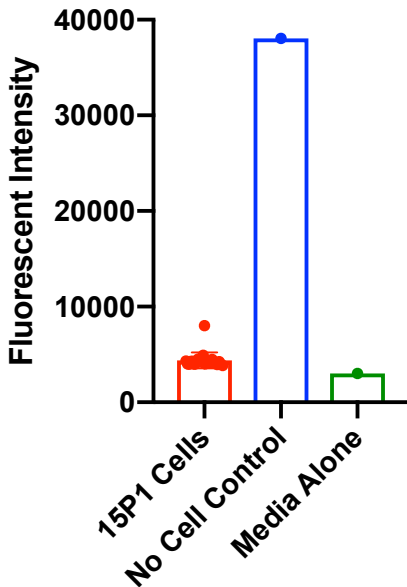

Supplement: S5 Fig — To confirm that the 15-P1 cell line could form a functional barrier, the monolayers were exposed to a GFP-Dextran after TEER values stabilized. The fluorescent intensity was measured from the bottom of the transwells after 2 hours of incubation and compared to a no-cell control and to media alone. Only the no-cell control transwell had GFP-Dextran move to the bottom of the transwell, indicating that the transwells had formed a functional barrier. (PDF) [file ppat.1008601.s005.pdf]

**A****ZIKV-NS3m**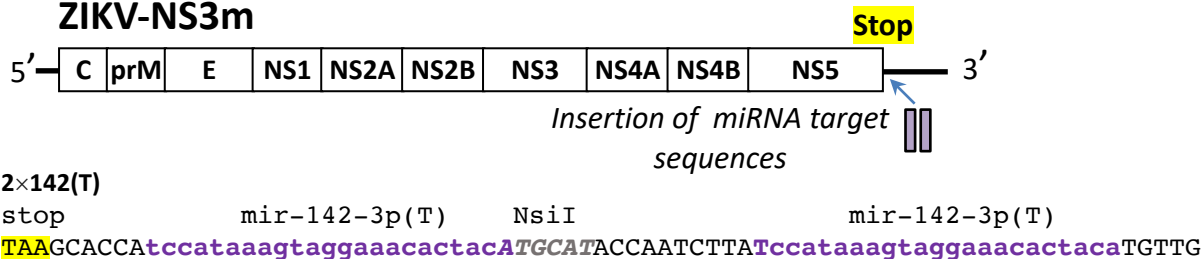**B**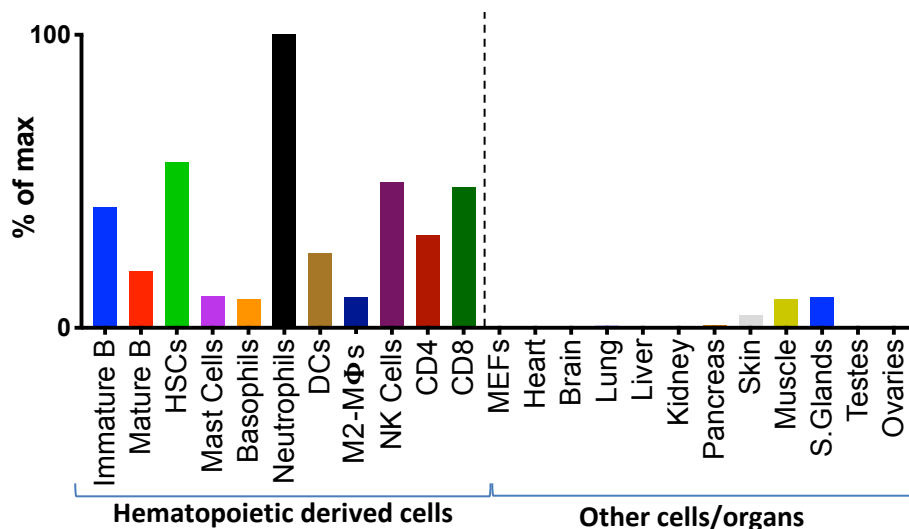**C**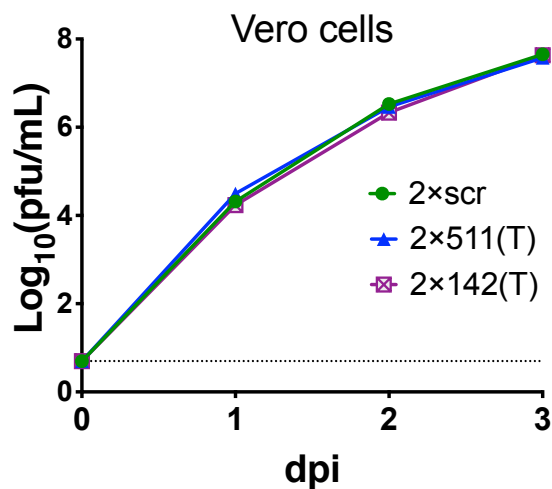**D**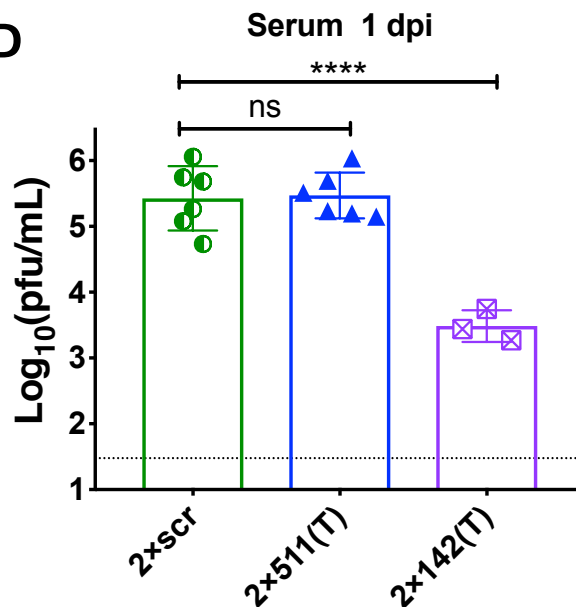

Supplement: S7 Fig — (A) Insertion of the mir-142-3p target sequences (highlighted in purple) into ZIKV-NS3m genome generating 2×142(T) virus. (B) Expression profile of the mir-142-3p in the selected cells and organs of mice. The graph was constructed based on deep sequencing data of the mouse miRNAs, which was reported earlier [38]. The expression profile is presented as a proportion of the number of reads for mir-142-3p in the cell/organs to the number or reads for this miRNA in the neutrophils, which has the highest expression level among all cell types. HSCs—hematopoietic stem cells, MΦs-macrophages, NK—natural killer cells, MEFs–mouse embryonic fibroblasts. C—Growth of 2×scr, 2×511(T) and 2×142(T) viruses in Vero cells after plasmid DNA transfection. D—Mean virus titer ± SD in the serum at 1 dpi. AG129 mice (n = 3–6 per group) were infected IP with 106 pfu of 2×scr or miRNA targeted viruses and bled at 1dpi [see Fig 2 for experiment details]. Differences between the virus titers were compared using one-way ANOVA (**** p < 0.0001; ns—denotes not significant). (PDF) [file ppat.1008601.s007.pdf]
