# Supplementary material for: Zika virus tropism during early infection of the testicular interstitium and its role in viral pathogenesis in the testes
Source: PLoS Pathog. 2020 Jul 2;16(7):e1008601. doi: 10.1371/journal.ppat.1008601 (PMC7331987; doi:10.1371/journal.ppat.1008601)
Supplement: S3 Fig — Adult AG129 mice (n = 7) were infected IP with 106 pfu of 2×511(T). Mice were bled at 1 dpi and sacrificed on 12 dpi. On the top—annotated sequence of the 5’ terminus of the 3’NCR for 2×511(T) virus. On the bottom–sequencing electrophoregrams of 2×511(T) virus genome isolated from the serum, brain and testes of mouse #5. Red arrows highlight the 3’-end of deleted sequence identified in 2×511(T) virus isolated from the testicular sample. Strikethrough sequence identifies 40 nt deletion in the testicular sample. (PDF) [file ppat.1008601.s003.pdf]

2×511(T)

stop mir-511-3p(T) mir-511-3p(T)

TAAGCACCA**ATcctgtcttttgctacacatt***ATGCAT*ACCAATCTTA**ATcctgtcttttgctacacatT**GTTGTC

Mouse #5

Sequencing direction ←

Serum 1 dpi (miRNA targets are stable)

Titer = 6.0 $\log_{10}$pfu/mL

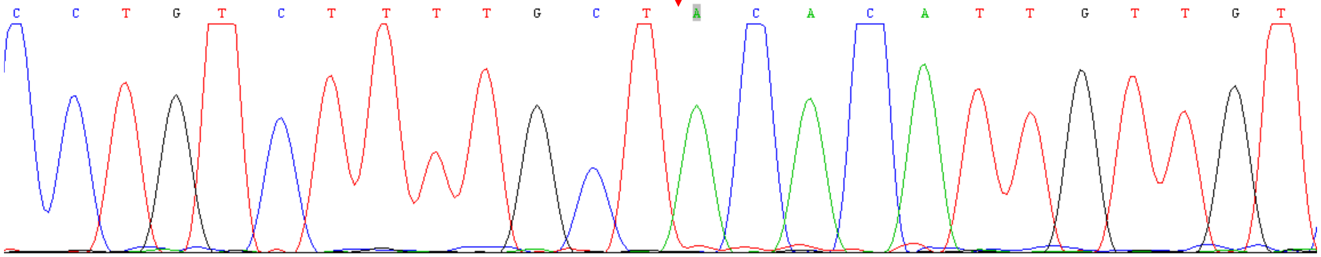

Brain 12dpi (miRNA targets are stable)

Titer = 5.7 $\log_{10}$pfu/g

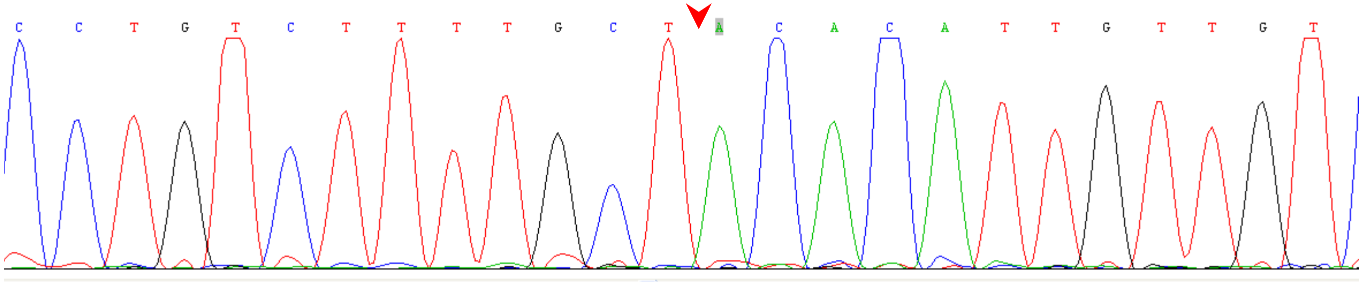

Testis 12 dpi (40 nt deletion in the region of miRNA targets insertion)

Titer = 5.6 $\log_{10}$pfu/g

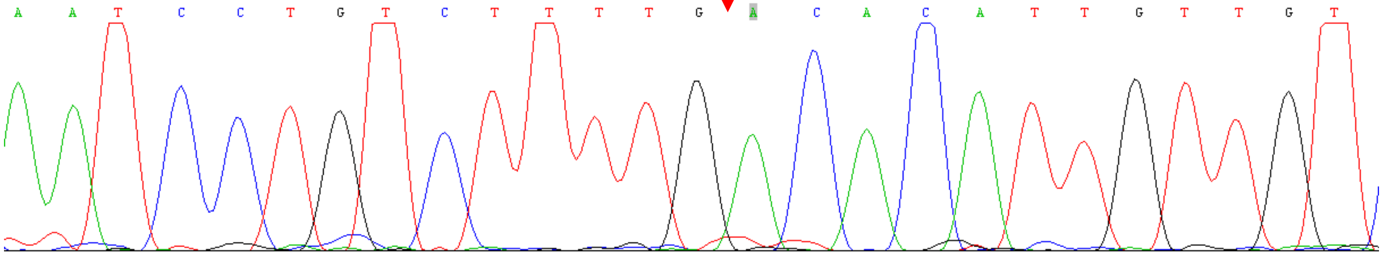

stop mir-511-3p(T) mir-511-3p(T)

TAAGCACCA**ATcctgtcttttg~~ctacacatt~~**~~*ATGCAT*ACCAATCTTA~~**~~ATcctgtcttttgct~~acacatT**GTTGTC

40 nt deletion
