# Supplementary material for: Zika virus tropism during early infection of the testicular interstitium and its role in viral pathogenesis in the testes
Source: PLoS Pathog. 2020 Jul 2;16(7):e1008601. doi: 10.1371/journal.ppat.1008601 (PMC7331987; doi:10.1371/journal.ppat.1008601)
Supplement: S6 Fig — (A) Schematic representation of C/3’NCR-511(T) virus genome. dCGR–duplicated capsid gene region. C-trn(50AA)–truncated C gene encoding 50 amino acids, which are cis-acting elements involved in the virus replication. *—open reading frame (ORF) shifting mutation [Fr Sh (+1)] denotes insertion one nucleotide in the C-trn. C-opt is a full-length copy of C gene, which is responsible for virion assembly. It contains synonymous mutations introduced in each AA codon (except ATG and TGG). 2A - autoprotease 2A from foot-and-mouth disease virus; the curved arrow indicates position of 2A protease cleavage site. Blue boxes indicate targets for mir-511-3p. (B) The annotated sequence of the dCGR. (C) The annotated sequence of the 3’NCR of C/3’NCR-511(T) virus. XhoI, KpnI and NsiI—restriction endonuclease cleavage sites that were used for cloning of the C/3’NCR-511(T) plasmid. (PDF) [file ppat.1008601.s006.pdf]

A

C/3'NCR-511(T)

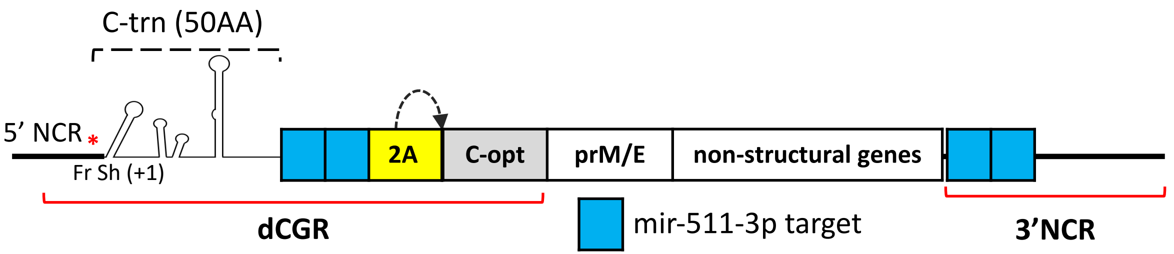

B

Insertion of targets for mir-511-3p into dCGR of the C/3'NCR-511(T)

```
                 +1A frame shift in truncated C gene (c-tr)
ATGAAAAACCCAAAAaAAGAAATCCGGAGGATTCCGGATTGTCAATATGCTAAAACGCGGAGTAGCCCGTGTGAGCCCCTTTGGGGGCT
                                                                  XhoI        mir-511-3p(T)
TGAAGAGGCTGCCAGCCGGACTTCTGCTGGGTCATGGGCCCATCAGGATGGTCTTGGCGATagCTCGAGgatcctgtcttttgctacac
           KpnI             mir-511-3p(T)                                   2A protease (FMDV)
attctgggactGGTACCgacatcctgtcttttgctacacattctggccacagtcctagggcaCTGAACTTCGACCTGCTGAAGCTCGCC
                              start codon of C-opt gene
GGCGACGTGGAGAGCAACCCTGGCCCTATG
```

C

Insertion of targets for mir-511-3p into 3'NCR of the C/3'NCR-511(T)

```
stop           mir-511-3p(T)    NsiI                mir-511-3p(T)
TAAGCACCAATcctgtcttttgctacacattATGCATACCAATCTTAATcctgtcttttgctacacatTGTTGTCAGGCCTGCTA
```
